# A Comprehensive Review of the Technological Solutions to Analyse the Effects of Pandemic Outbreak on Human Lives

**DOI:** 10.3390/medicina58020311

**Published:** 2022-02-18

**Authors:** Ishwa Shah, Chelsy Doshi, Mohil Patel, Sudeep Tanwar, Wei-Chiang Hong, Ravi Sharma

**Affiliations:** 1 Department of Computer Science and Engineering, Institute of Technology, Nirma University, Ahmedabad 382481, Gujarat, India; 18bce218@nirmauni.ac.in (I.S.); 18bce062@nirmauni.ac.in (C.D.); mohilpatel21@gmail.com (M.P.); 2 Department of Information Management, Asia Eastern University of Science and Technology, New Taipei 22064, Taiwan; 3 Centre for Inter-Disciplinary Research and Innovation, University of Petroleum and Energy Studies, P.O. Bidholi Via-Prem Nagar, Dehradun 248007, Uttarakhand, India; ravisharmacidri@gmail.com

**Keywords:** COVID-19, human lives, social, healthcare, behavioural

## Abstract

A coronavirus outbreak caused by a novel virus known as SARS-CoV-2 originated towards the latter half of 2019. COVID-19’s abrupt emergence and unchecked global expansion highlight the inability of the current healthcare services to respond to public health emergencies promptly. This paper reviews the different aspects of human life comprehensively affected by COVID-19. It then discusses various tools and technologies from the leading domains and their integration into people’s lives to overcome issues resulting from pandemics. This paper further focuses on providing a detailed review of existing and probable Artificial Intelligence (AI), Internet of Things (IoT), Augmented Reality (AR), Virtual Reality (VR), and Blockchain-based solutions. The COVID-19 pandemic brings several challenges from the viewpoint of the nation’s healthcare, security, privacy, and economy. AI offers different predictive services and intelligent strategies for detecting coronavirus signs, promoting drug development, remote healthcare, classifying fake news detection, and security attacks. The incorporation of AI in the COVID-19 outbreak brings robust and reliable solutions to enhance the healthcare systems, increases user’s life expectancy, and boosts the nation’s economy. Furthermore, AR/VR helps in distance learning, factory automation, and setting up an environment of work from home. Blockchain helps in protecting consumer’s privacy, and securing the medical supply chain operations. IoT is helpful in remote patient monitoring, distant sanitising via drones, managing social distancing (using IoT cameras), and many more in combating the pandemic. This study covers an up-to-date analysis on the use of blockchain technology, AI, AR/VR, and IoT for combating COVID-19 pandemic considering various applications. These technologies provide new emerging initiatives and use cases to deal with the COVID-19 pandemic. Finally, we discuss challenges and potential research paths that will promote further research into future pandemic outbreaks.

## 1. Introduction

As humans have progressed through the years, and there has been a change regarding how individuals respond to various situations and how society responds to the actions of an individual. Understanding the significance of human lives requires a thorough analysis of how each life can impact the environment and the community. Especially in the case of calamities or adverse situations, as in COVID-19, every human response matters. Social relations establish a link of familiarity towards the community and the lack of them alienates the individual. When a scenario such as the current one arises, this can have harmful repercussions. Human behaviour, even on an individual level, always has further influences on a larger scale. This is because humans are connected and hence every action affects the group they are connected with.

The influence of human behaviour can be seen as a consequence of the person’s mental activity. Beliefs, trends, and valuables affect a person’s behaviour regarding how seriously one views privacy and connections. Thoughts and explanations of the mind often provide acute reasoning behind human actions. Further, human actions have since long impacted the people and the surrounding environment. Furthermore, actions depict the development of an individual. Thoughts, beliefs, and trends that hinder human development are harmful to society, as they obstruct society from utilising human potential. On an individual level, a hindrance to human development affects the individual’s ability to earn a proper livelihood, affecting their mental health and creating a vicious cycle of ill effects.

For most individuals, the surrounding environment often includes workspace. Their behaviour, thus, has an impact on the work environment and their co-workers. On a professional level, this can result in a loss of productivity over a while. Elements of psychology, particularly social psychology, such as responsibility, motivation, teamwork, management, supervision, and leadership, amongst others, are pillars of a healthy work environment.

Human responses are repeatedly perceived as guidance of the education individuals receive. Education and sufficient knowledge regarding local and global conditions and changes are essential to avoid panic on a large scale. Lack of the same can create mass hysteria as a result of a sense of facing the unknown. To calmly handle any large-scale crisis and help society overcome it, humans need to exercise their adaptation skills. It is an innate skill inside each human which, only when honed, can benefit the community. The faster humans adapt, the better the situation can be handled. Based on how well individuals adapt to the changes and respond to the factors affecting the changes in livelihood, economic and financial changes may be triggered.

It can, thus, be said that human behaviour and the actions influenced by this behaviour are susceptible to a large number of factors ranging from culture, age, nationality, and surroundings to education, self-awareness, and knowledge. Since such effects on behaviour can result in actions that impact society, it is important to consider establishing a taxonomy that provides a basis for the study of human psychology, behaviour, and actions of interpersonal relations.

### 1.1. Motivations and Contributions

#### 1.1.1. Motivations

The motivations behind this study are as follows.

- Existing literature provides a vertical classification of one of the aspects of life affected by COVID-19.
- Even within the existing literature, most articles only focus on the psychological, academic, healthcare-based, and economic impact of COVID-19 on human lives. Few materials are available on the behavioural and social impact, fewer on the interpersonal impact.
- Majority of the available research focuses on developments in one particular field to combat the situations caused by COVID.
- Thus, we felt the need to provide for a combined review of the effect of COVID-19 in different aspects of human life and the different solutions proposed for mitigating the problems arising in these aspects.

#### 1.1.2. Contributions

The major contributions of this paper are as follows.

- Through this review, we present a horizontal classification of the different aspects of human lives affected by COVID-19 in the form of seven major aspects and how severe the effects are—both positive and negative.
- We provide a comprehensive analysis of the different technological domains involved in the suggested or implemented solutions to COVID-19 and the aspects affected adversely by it. The analysis is conducted considering both the general outlook of human life as well as the individual seven parameters.
- Lastly, we discuss the various challenges, open issues and future research directions in the domains largely analysed.

### 1.2. Review Methodology

This section discusses the detailed methodology to conduct this review.

#### 1.2.1. Review Plan

To carry out a systematic study and lay down a structured approach, this review section constitutes the planned format of review planning, research questions to obtain a clear grasp on the review statement, the data sources relevant to the statement, search criteria in effect during the literature review, the criteria of inclusion and exclusion, as well as the quality evaluation. Numerous existing literature sources, including news articles, international organisation updates, and publications, were identified and referred to for the study. The included sources have been checked for quality before considering this study.

#### 1.2.2. Research Questions

To gather a clear understanding of the review statement, the authors have collected and ascertained the literature relevant to the statement in terms of both impact of COVID-19 on the aspects of human lives and the technological solutions that aided the impact mitigation. Table 1 shows the possible research questions along with the objectives were identified to write this review.

#### 1.2.3. Data Sources

A comprehensive survey requires a large number of reputable and reliable data sources. Our resources include journals, articles, and books, from the medical arena to technical details. For finding the existing literature on the impact of the pandemic and how technologies helped to tackle the issue, we preferred only standard peer-reviewed journal databases such as IEEEXplore digital library, Science Direct journals (Elsevier), ACM Digital Library, Pubmed journals, and Springer link, as well as the electronic data sources recommended by the authors. Other data sources include technical publications (published on the IEEE, Elsevier, and Springer platforms), reports (Government reports, WHO reports), prediction agency websites, and online literature (pharmaceutical blogs, technical blogs, news articles) that are relevant to the issue suggested in a comprehensive survey.

#### 1.2.4. Search Criteria

**Figure 1:**
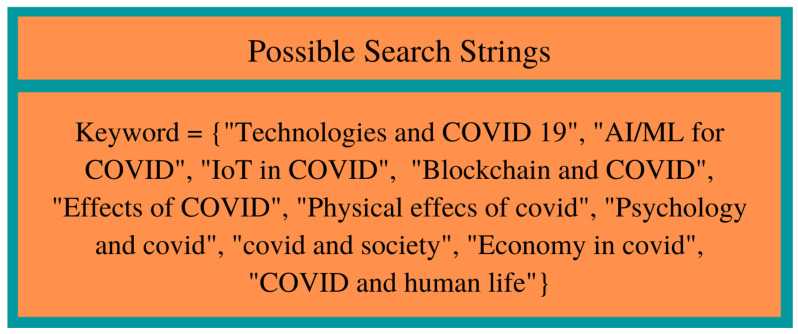
Search strings.

The search criteria in this survey include keywords such as “Technologies and COVID 19”, also the combination of technologies such as “AI, ML, IoT, AR-VR, Blockchain” along with “Social, Economic, Psychological Behavioral, Interpersonal, Healthcare, or Pandemic”, and other related keywords referred for the search criteria in the comprehensive survey, as shown in Figure 1. Many research papers/articles have search strings that are not found in the title or abstract of the publication; in these cases, a manual search was conducted.

#### 1.2.5. Criteria of Inclusion and Exclusion

**Figure 2:**
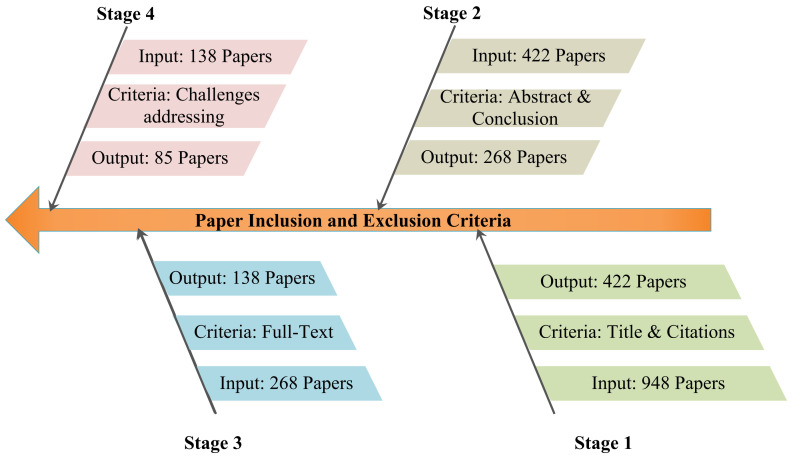
Criteria of inclusion and exclusion.

Because one of the survey’s main goals is to show how technology has aided in the COVID-19 epidemic, the search string “COVID 19” and “Technologies” will often return irrelevant results, making filtration difficult. To avoid this, follow the search criteria outlined in Section 1.2.1. The most recent and relevant publications from 2020 and early access articles are included in this poll to make it more appealing and impactful. Various survey articles, technical patents, books, news reports, blogs, and other resources are included for a broader perspective. Figure 2 depicts the filtering of publications based on relevance and other characteristics. Based on the title, abstract, entire paper, and research, this filtration is divided into numerous steps. Finally, we found a few relevant publications and focused on those with high citation counts.

#### 1.2.6. Quality Evaluation

In this part, the quality of the discovered publications has been assessed using the guidelines provided by professional organisations such as the Database of Abstracts of Reviews of Effects (DARE) and the Center for Reviews and Dissemination (CRD). The quality questions are listed in Table 2, and they were used to choose the research publications.

### 1.3. Scope of the Review

Table 3 shows the different research articles and conference proceedings published till date that involve papers related to COVID-19 and its impact on human lives [1,2,3,4,5,6,7,8,9,10,11,12,13]; however, as observed, most of these papers focus either on one aspect, or involve region focused research outcome. Goodwin et al. [1] talked about the impact of COVID-19 and the quarantine imposed by majority of the countries on interpersonal relationships between people. The article also talked about the psychological distress caused amongst the public as a result of the sudden disruption of routine life. The article involved a study conducted amongst people in China to analyse the change in different interpersonal relationships. Chamola et al. [2] presented a comprehensive review on COVID-19 and the role different technologies played in aiding COVID-19-based mitigation and management. The review article covered information regarding COVID-19 and its impact on the global economy and different industries. Given that its main purpose was to discuss the use of different technological advancements in managing COVID-19, there was an extensive analysis towards the different COVID-19 related advancements and their usefulness in aiding the testing and treatment. Arora et al. [3] on the changes brought in the education model around the world, as a result of the pandemic. Socio-economic impact of COVID-19 was the focus for the authors of [5], whilst Ozili et al. [6] concentrated on the economy and global spillover. Psychological Impact of COVID-19 was the emphasis of the articles by Serafini et al. [9] and the authors of [11].

As seen, different authors focused solely on few of the different aspects. This review aims to consolidate the different aspects of human life and the diverse impact of COVID-19 on these aspects. At the same time, the authors also discuss how the technological advancements have aided various human aspects during pandemic mitigation. Presenting a comprehensive review that covers the different aspects and displaying that technological aids can help in mitigating the disruption observed in these aspects, is the main objective behind this review.

### 1.4. Organisation and Reading Map

**Figure 3:**
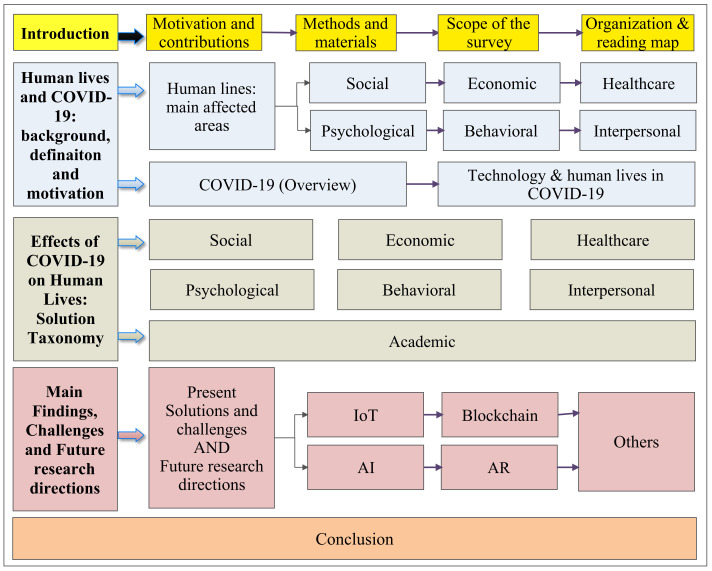
Structure of the review article.

The survey’s structure is depicted in diagrammatic form in the Figure 3, which demonstrates the survey’s horizontal nature of information covered. The rest of the paper is organised as follows: the background of the COVID-19 pandemic is discussed in Section 2, and Section 3 provides a full explanation of human life characteristics, such as social, healthcare, economics, psychology, behavioural, interpersonal interactions, and academics, as well as how the epidemic has influenced them. Section 4 examines the utilisation of key technologies such as the Internet of Things (IoT), Blockchain, Artificial Intelligence (AI), and Augmented Reality (AR)/Virtual Reality (VR) to combat COVID-19, as well as the problems they pose and future research opportunities. The final section presents the conclusion and recommendations.

## 2. Human Lives and COVID-19: Background, Definition and Motivation

Table 4 shows the relative comparisons of existing surveys that we considered after conducting a thorough literature review. During our literature review, we discovered a lot of literature focusing on the vertical arena, whether it is interpersonal, psychological, academic, or economic aspects of human life in a pandemic situation. We try to include all horizontal aspects of human life that are affected during the pandemic in this review paper. Table 1 attempts to present the survey’s objective, key contribution, and limitations in an organised and concise manner. Following a comparative analysis of the literature, we identified seven aspects of human life impacted by COVID-19: healthcare, economic, social, psychological, behavioural, interpersonal, and academic.

Human beings are shaped through changes in various aspects of their lives, and recent months have seen changes that have thrown off the previous balance maintained within these aspects. The effects on these aspects have been briefly discussed in this section.

### 2.1. Human Lives: Main Affected Areas of Human Lives

#### 2.1.1. Social

Human beings are inherently social creatures and their interactions with fellow individuals are important for maintaining decorum in their lives. The shift in the daily routine has involved many changes, including restrictions on social interactions, public gatherings, celebrations, group tasks, educational gatherings, and private meets [14]. These social events or activities have a level of influence on human lives.

Cultural gatherings are often seen as a common activity in social customs and often helpful in connecting people. Religious assemblies or rituals, carried out daily, are also a meeting ground under social customs. These religious activities occupy a significant part of most human lives. Even the silent mutual presence of strangers or acquaintances in common surroundings is a social interaction that humans are accustomed to. A sudden stop to this social expectation of an individual’s daily life can have colossal repercussions.

#### 2.1.2. Economic

Given the increasingly threatening health crisis, an immediate solution in terms of a complete lockdown was put in place. Private businesses [15,16], such as retail shops and godowns, were majorly shut down. Only a few shops such as provision stores, dairy stores, and medical stores remained working. In the case of banks and private, multinational companies, employees’ hours were reduced, or their presence was expected to be reported online instead of physical appearance.

Due to the sudden decision, several people were stuck out of their hometown and away from jobs. Many of them were unfamiliar with their environment. Labourers were also unable to return to their villages and urgent travel options were also blocked. Thus, people faced a lot of economic problems and the nation also faced a crisis [17].

#### 2.1.3. Healthcare

Human body and health often hang onto a delicate thread that requires great care and attention [18]. When physical health is at risk, drastic and abrupt measures might be taken. As in the case of the recent pandemic, the rapid spreading of the contiguous disease caused various speculations about public health, especially the children and the senior citizens.

The youth might have a higher chance of recovery [19]. However, this certainly does not conclude that they have a lower chance of being infected. Further, major households have either children, or older adults, or both. The virus could be transmitted to them through family members. Thus, it was necessary to ensure that venturing out was not an option but rather the last choice and minimised. To minimise interactions, only businesses involved in products essential for daily use functioned majorly [20]. Even then, restrictions were placed to cut down physical contact as much as possible. With the restrictions in place, a shift to the virtual world occurred [21]; however, other health issues still prevail and there have been both positive and negative effects on overall physical health.

#### 2.1.4. Psychological

Human beings are said to be fragile psychologically. Fluctuations of emotions [22] are often closely intertwined with mental issues. When physical surroundings around individuals differ, it also impacts their minds [23].

Psychological responses may differ from individual to individual or from group to group. In situations of sudden changes, these responses might be more turbulent than otherwise. Moreover, with the unexpected shift in other major aspects of daily lives, social, economic [24,25], and healthcare-based conditions, there are bound to be drastic effects on mental health [26] as well.

Further, with the general focus swaying towards the spread of the pandemic and multiple endeavours being centred curbing it, we neglect the fact that other facets might prevail.

#### 2.1.5. Behavioural

Human behaviour is often strongly relates to a person’s surroundings and evolves due to an individual’s mental state. An individual’s behaviour is also influenced by the behaviour of other individuals in the vicinity. There might also be an abrupt change in human behaviour based on the response received [27]. Responses can also have delayed effects, in which case, the behaviour might be witnessed by other individuals who were not responsible for the previous response. This can further influence their behaviour.

Thus, human behaviour might result from a chain of actions of more than one individual, or directly influence the people involved [28].

#### 2.1.6. Interpersonal

Humans are social animals, so interpersonal relationships are often seen as the pillar for the creation of human life. Interpersonal relationships refer to the connections or interactions that humans have with each other. Interpersonal relations are interconnected to an individual’s mind, body, and spirit. During each phase of life, the interaction plays a very crucial role, irrespective of age. For children, it is important to establish good communication with teachers, friends, and family [29]. For the working class, proper workplace relations boost the quality and pace of work. Due to COVID-19, interpersonal relations have drastically been affected by the complete lockdown observed throughout the world [30]. The lack of communication leads to alleviation in anxiety, stress, loneliness, fear, and psychological disturbances, which indirectly lead to physical health deterioration [31]. Until the launch of the proposed vaccine, non-pharmaceutical interventions were the only ways to prevent the worsening effects of the virus.

Hence, since interpersonal relations touch all the elements of the body, mind, and spirit and majorly leave positive effects such as mental wellness, stress reduction, alleviation in physical healing, and reducing the recovery time, they need to be established differently in the pandemic situation.

#### 2.1.7. Academic

There are petrifying impacts of COVID-19 in the world, to reduce the spread of the pandemic certain steps are taken and one of them being complete lockdown due to that all educational institutes all over the world are closed. As a consequence to which, learners ranging from pre-primary school going to postgraduates are affected. There is a sudden shift of traditional education methods to digital education without prior knowledge regarding the same [32]. Hence, there was no time to analyse the potential risks that this shift can bring. The effects of this shift vary in different countries as some countries are developed and some are developing countries, developing countries deprived of a strong backbone structure for conducting online studies resulting in facing frustration, stress, and anxiety in students [33]. Many entry-level exams have adhered and amendments are taking place. Thus uncertainties in educational policies result in mental turmoil [34]. Education is one of the most important factors for the growth of any nation, so if the growth rate slows down due to the unavailability of technological resources, it will affect the nation’s growth rate, which in turn affects the economy of the country. Hence factors are interrelated, leading to a boom in mental stress in a wide range of communities.

### 2.2. COVID-19

#### Overview of COVID-19

COVID-19, broadly known as the coronavirus disease, is an infectious disease that generally affects the upper or lower respiratory tract [35]. Checking, it stands for (CO) Corona (VI) Virus (D) Disease 2019. Transmission of this virus generally occurs through direct or indirect contact with a person carrying the SARS-CoV-2 strain.

The SARS-CoV-2 is a strain of the coronavirus, which is responsible for the COVID-19 disease, and hence, has contributed to the pandemic situation [36]. SARS refers to severe acute respiratory syndrome, which is often linked to some types of the common cold. The SARS-CoV-2 strain, the latest strain, is often called ‘novel coronavirus. Its symptoms can range from loss of smell and taste, fever, cough, and shortness of breath in mild cases, to pneumonia and breathing difficulties in extreme ones [37]. By itself, the death rate of the disease had been fairly low; however, its spread has been exponential due to the relatively easy transmission. It may be transmitted directly through the eyes, nose, and mouth, or transmitted through contaminated surfaces. This happens because, without the use of any disinfectant, the virus can stay suspended in the air or on surfaces for long periods, ranging from three hours to three days and further [38].

Moreover, based on various reports, it has been observed that due to insufficient resources for the treatment of the disease on a large scale, patients of COVID-19 have often been faced with inadequate facilities and hence, have contracted other complications as a result of a weak immune system, courtesy of the COVID-19 virus.

Hence, to avoid further spread of the disease, a lockdown ensued in various parts of the world. This decision came as an apparent mode of action to minimise contact between people and control or stop the transmission. Social distancing is the best preventive measure for the virus. According to WHO, the minimum social distance to avoid droplets of coughing, sneezing, talking, and singing is 1 metre or 3 feet [35]. At the same time, the United States Centres for Disease Control and Prevention (CDC) recommends 2 metres or 6 feet of distance [35].

After the uplift of the lockdown, to mitigate its effects, people had been asked to follow other preventive protocols that include washing hands frequently with soap and water, using masks while going out, avoiding close contact, looking out for symptoms of the disease, and seeking medical care in case of symptoms [35].

However, 2021 has seen new variants of the SARS-CoV-2, bringing in the second wave of COVID-19 in many parts of the world. These variants have proved to be deadlier than the previous one and have increased death rates.

The governments are mitigating the same through improved testing and healthcare facilities and vaccination drives.

### 2.3. Technology and Human Lives in COVID-19

In the pandemic situation of COVID-19, when there is an absence of proper vaccine or therapy, it is important to incorporate technologies to obtain different solutions with varying degrees of success. Countries use these strategies for early detection, testing, surveillance, contact tracing, implementation of lockdown measures, monitoring mental health, running the business, academic purposes, and many more. Integration of Artificial Intelligence (AI) into the Internet of Things (IoT), Blockchain, and AR/VR is one of the most effective options for assisting this method. Though AI aids in data analysis, blockchain technology offers a stable framework in which to develop a reliable, distributed method of sharing and storing data, IoT collects data, and AR/VR used for virtualisation and visualisation [39].

Researchers are investigating each possible way to fight against the pandemic. Technology advancements have brought many successes into our daily lives, and they have also aided people in an extremely difficult fight against COVID-19. This integration lowered the mortality rates, reduced the spread of COVID-19, reduced mental stress, and changed people’s behaviour. There are many discernible changes in daily human lives due to technological revolution such as people started shopping through online apps rather than going to malls or stores, the offices shifted their work on online technological platforms, malls installed face scanners, body temperature measuring equipment, doctors use advanced tools and models to test and cure COVID-19, students started using open-source study materials and online-meets for study purposes, etc.

Table 5 discusses various solutions in brief and also, there is mention of which parameter of human life it solves. More detailed discussions about technological solutions, their impacts, challenges, pitfalls, and their future scopes are presented in Section 4.

## 3. Effects of COVID-19 on Human Lives: Solution Taxonomy

The social, economic, psychological, behavioural, academic, interpersonal, and healthcare-based aspects of human life, as influenced by COVID-19 and the lockdown, have already been briefly touched upon in Section 2. Table 2 further emphasises the relevance of these aspects to the impact of COVID-19. Detailing the same, the effects and their dependency upon each other have been detailed in this section.

### 3.1. Social

Society, in its essence, has a large amount of influence on an individual’s life. Be it an individual’s mentality or his lifestyle, and societal decisions often prompt his decisions. So, when this influence is minimised all of a sudden, the social aspect of human life is greatly disturbed.

One of the major aspects of the lockdown period is social distancing. Social distancing involves minimal interactions with fellow human beings or even with the familiarised environments beyond the residence. Routinely, most people have various social interactions—be it friends, family, co-workers, fellow passengers on public transport, or local shopkeepers that provide daily essentials.

This provides a sense of familiarity, which roots the individual. With the restrictions on these interactions, individuals have lost this sense of familiarity and belonging in the surrounding environment. This absence can lead to a change in people’s behaviour such that they may no longer be socially compatible with their groups and surroundings. The ceasing of religious gatherings, celebrations, and activities has also altered people’s faith and beliefs. Further, according to reports, to re-establish the belief system, people had participated in large-scale religious gatherings despite the guidelines that stated otherwise. This had, in turn, led to an increase in the spread of the pandemic, defeating the very purpose of lockdown. After the lockdown was lifted, these gatherings increased in the magnitude of people joining [73].

Moreover, to reconnect with their culture, to return to a sense of familiarity, and to minimise the expenses, a lot of people decided to migrate to their hometowns and villages, particularly migrant workers [74,75]. For some, this had a better outcome as the native regions were relatively safe and the travel was also easier; however, for various others who had no proper transport options available, this became a difficult journey.

With the increasing cases and extended lockdown, social unrest mounted in different parts of the world, especially for the elderly. To console the public and relieve the unrest, digital communications were stressed upon, particularly in places such as nursing homes. The children and the elderly, now unable to leave the house for fear of contracting the disease, shifted to e-commerce and communication [76].

**Figure 4:**
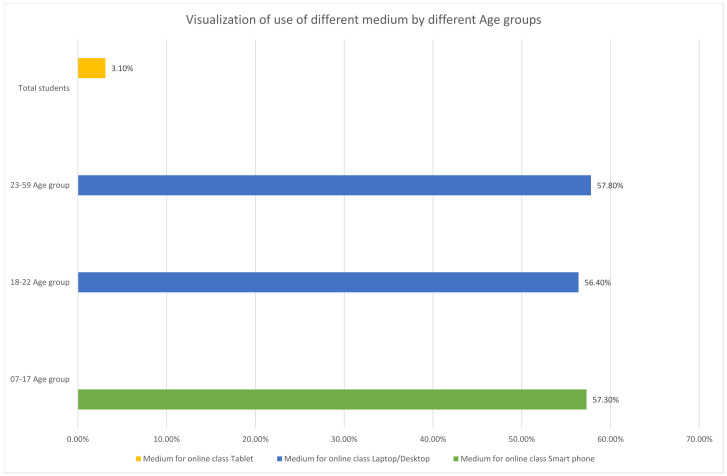
Visualisation of use of different gadgets by different age-groups [81].

The use of social media applications, and digital devices in general, also increased, not just for the youth, but for all age groups [77,78]. Figure 4 will help to visualise the preference of different gadgets for online medium such as smart phones, tablets, desktops, and laptops among various age group of people. Under the lack of outdoor activities, people shifted to innovative ways to spend free time at home. Birthday celebrations turned virtual. Cooking shows and videos saw an increase in the number of viewers. Schools and work moved to virtual platforms. Sales of video streaming applications such as Amazon Prime Video, Netflix, and Hotstar boomed, among various other such scenarios [79,80]. The one concern that remains is whether people shall eventually revert to their daily lives, the routine before the pandemic, and whether humans as social creatures would turn into social recluses.

### 3.2. Healthcare

Health is the most important aspect of human life, as, without good health, all the other aspects of human life will be affected [82]. Especially with the pandemic, human health is in jeopardy [83]. To protect the public from the rapid spread of the COVID-19 virus, various countries issued a lockdown. Social distancing was to be maintained, and venturing in public places was restricted. Children and senior citizens were not allowed to leave the house, keeping their fragile immune systems in mind. Various nations brought up different measures to deal with the crisis [84]. Countries such as Singapore and South Korea adopted digital means of testing [85]. European countries such as Germany arranged for large-scale testing and proper utilisation of the availability of ventilated beds, amongst other resources [86]. This, along with healthcare facilities for the patients, helped secure public health overall.

Despite this, the resources required to handle the pandemic compared to the affected population on a global scale were inadequate. At the same time, with the focus shifted to COVID-19, its prevention, treatment, and the patients, other deadlier healthcare areas, associated with cardiovascular, pulmonary, metabolic diseases, and cancer, were neglected and vulnerable to the virus due to the already weak immune system [87,88]. Regular cough, cold, and other infections of smaller degree were also difficult to investigate with various clinics shutting down. People avoided larger hospitals for fear of becoming infected [89,90]. Due to poor facilities in certain hospitals, patients of other diseases contracted COVID-19 from direct or indirect transmission via the hospital staff.

Certain COVID-19 patients also became infected with more harmful diseases such as dengue and swine-flu.

Furthermore, the economic crisis and shutting down of workplaces, faced by the countries and individuals, resulted in a loss of income for many people. This made it difficult for them to provide nutritious food to the families, resulting in malnutrition [91]. On the other hand, cases of weight gain and obesity also increased with the lack of physical activity [92]. This resulted in those individuals being at a higher risk of contracting the coronavirus or at a higher risk of developing of diabetes or cardiovascular diseases in the future.

Moreover, neglect in the healthcare of pregnant women could result in an increased number of miscarriages or maternal deaths [93]. In the case of maternal malnourishment, the child might be born prematurely, resulting in complications. In successful births, the child would still have a high chance of getting infected, which would drastically lower the chances of survival. The mother would also be at a high risk of getting infected, which would affect the health of the child and potentially the rest of the family through direct transmission.

Collapse of the public health care system was a serious possibility under the conditions, which would have led to a radical loss of human lives.

### 3.3. Economic

The implications of COVID-19 can be witnessed in a thoroughly disrupted economy in the span of a few months. All sectors of the economy have been heavily affected, and several businesses are on the verge of collapsing.

With most businesses, hotels and restaurants being closed, the demand for raw materials of petroleum, and mining industries, among others, has been low. The majority of the people employed in these industries have few other livelihood alternatives. It has been very difficult for them to sustain their families under the economic uncertainties of the pandemic.

Among manufacturing industries, those involved in the manufacture of medicines, daily essentials, fruit and vegetable markets and dairy products have not faced any decline in sales. Some have even produced more goods as an indirect result of panic buying [94]. Organic farm products have been more in demand since the pandemic. According to ReportsWeb, it was expected that the global organic farming market would grow at a compound annual growth rate (CAGR) of 8 percent, from approximately 88.79 billion dollars in 2019 to 95.90 billion dollars in 2020. Many countries have had stable growth due to the COVID-19 outbreak [95]. The expected new total aftermarket recovery is 131.55 billion dollars in 2023 at a CAGR of 11.11 percent.

Farmers have, thus, established direct contact with customers and gained a means of sustenance; however, in the case of certain commodities, panic buying has also led to ‘the bullwhip effect’ [96]. At the same time, sales of other seasonal or luxurious commodities have declined. There is a proposed decline in the sales of luxury goods by 18 percent in 2020 as a result of COVID-19 [97]. The automobile industry has taken a hit from the pandemic with a sharp decline [98].

Surprisingly, sales of certain goods have seen an unexpected rise. This has resulted in greater demands for manufacturing. The sales of used cars have gone up in India [99,100]. Sales of comfortable footwear, including flip flops and Crocs, have also risen. NewsBytes reported an increase in sales of Crocs and other live-at-home footwear while reporting a 15 percent decline in overall shoe sales in the United States [101]. According to the Economic Times, online searches for Crocs and other such live-at-home footwear had increased 53 percent since June 2020 and the demand for flip flops increased by 89 percent month-on-month abroad. Sales of flip flops, sandals, and slides reached up to 80% of the pre-COVID levels in India as well [102].

**Figure 5:**
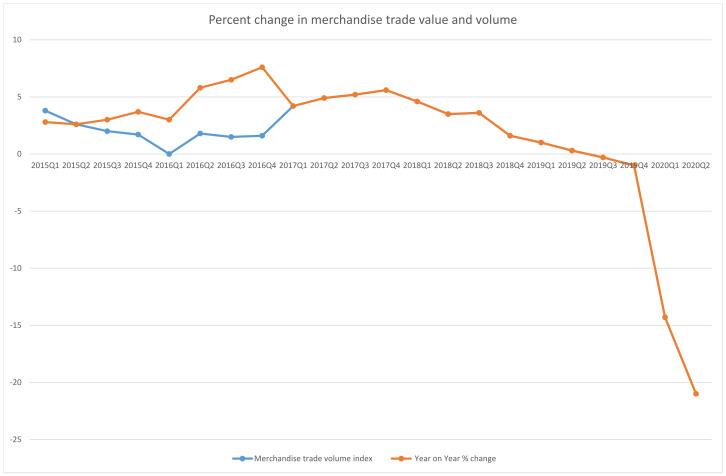
Percent change in merchandise trade value and volume [104].

The service sector has seen ups and downs in these past months. While the IT sector has had little to moderate impact with ‘Work from Home’ available, some other industries have been highly affected. The tourism and transportation industry, aviation industries, cosmetics industry, and the sports industry are among the few that have suffered the most. The tourism sector alone faces an output decrease as high as 50 percent to 70 percent [103]. As stipulated by international organisations such as WTO (World Trade Organization) and EOCD (Organisation for Economic Growth and Development), since the last financial emergency of 2008–2009, COVID-19 can be considered the largest threat to the global economy. In terms of both trade merchandise value and volume, world trade has also witnessed a decline. Figure 5 is the graphical representation of percentage change in merchandise value and volume. This contraction, of 14.3% decrease by value and 21% by value as seen in the second quarter of 2020, is sharper than the 10.2% drop seen between the end of 2008 and beginning of 2009 [104].

However, while the above-mentioned industries have suffered, the demand for digitally available goods and services has increased. Based on the WTO e-commerce report of 4 May 2020 [105], E-commerce is essential to consumers, and this has become clear under the pandemic. With the limited availability of goods and services locally, consumers have shifted to e-commerce. E-commerce makes it easier for sellers and service providers to connect to the customers while following the safety guidelines at the same time. Digital gaming is one such service that has prospered in the pandemic [106], video conferencing [107], another. With theatres closed, video streaming apps have also seen a surge, as briefed previously. To provide these digital products and services and facilitate living in times of COVID-19, other requirements have sprung up, which have paved the way for various digital startups. Ranging from healthcare-based applications for contact tracing to delivery-based applications that provide delivery facilities for daily essentials, new necessities have brought forth many new digital solutions. Considering individuals, this has provided an opportunity for many freelancers as well as part-time employees.

Whilst COVID-19 has impacted countries and organisations, and it has also impacted the public at an individual level. Most low-income and below-poverty-line families have been heavily affected, their means of livelihood at a standby. Construction workers, helpers and labourers had very little work availability. In India, this resulted in many of them migrating large distances to their villages on foot. On the other hand, many individuals have stuck away from their families and livelihoods, with no means of returning. Many were stuck in a different place, altogether, as tourists, with limited monetary facilities available. This affected their mental health as well as their financial situation. Amongst other employees working for organisations, many faced salary cuts and loss of jobs. This left them uncertain about their means of income. Most businessmen, especially hotel and restaurant owners, faced another dilemma as their businesses were shut down or faced losses throughout the last few months. Thus, COVID-19 has deeply influenced the economic aspect of human lives.

### 3.4. Psychological

Psychological changes in humans have always been closely interconnected with the changes in their surroundings and routine lives. A drastic change such as COVID-19 and its impacts is bound to have a deep psychological impact on the human mind. Changes seen in the social and cultural, economic, academic and healthcare-based aspects, amongst others, have disturbed the mental balance of the public.

The times of COVID-19 have forced people to stay indoors. While necessary to minimise the spread of the pandemic, this decision has caused psychological stress for many [108]. The reduced social and interpersonal interactions with people, other than over social media, have resulted in loneliness and isolation. For some, there has also been an air of suffocation. Further, with work either on standby or via digital platforms, boredom and lethargy have taken the front seat. Lack of motivation has also reduced the work efficiency of the working population.

Most importantly, though, the driving factor behind the psychological stress has been fear. With large amounts of fake news circulating social media and few trustworthy media outlets, the fear of fake news has made people afraid of seeking out news altogether, resulting in a lack of awareness. On the other hand, believers of the incorrect news have been burdened with the wrong information, creating unnecessary panic.

Moreover, with the rapid transmission of COVID-19, people fear the uncertainty of their lives. The fear of being infected has caused anxiety and panic attacks among the masses. Those families that have witnessed the effects of being infected first-hand feared death in the family. With limited medical facilities available for COVID-19, compared to the numbers infected, patients suffering from the virus also fear contraction of other diseases due to mismanagement, sanitation availability, and lack of proper facilities. In addition to patients of COVID-19, patients who suffer from other diseases and have been admitted to medical facilities would fear contracting the COVID-19 virus. All of this has a major impact on mental health, which hinders the speed of recovery. Other than that, for patients with mental illnesses before COVID-19, their grievances have increased. There are high chances that patients who are already previously diagnosed with psychosis fail to comply with the social distancing norms and personal care and hygiene requirements necessary during an outbreak of infectious disease; thus, representing a huge potential risk to the entire community and even the mental health clinicians [8]. It is not just the patients that have faced psychological meltdown. The health workers, police officers and others who are involved first-hand in the pandemic themselves fear the huge risk they are at.

In addition to this, with the economy in turmoil and companies cutting down on costs, the working personnel fear the loss of jobs and income. Businessmen also fear huge losses as a result of the pandemic. So, monetary issues, or rather, the possibilities of monetary issues, have instilled the fear for basic livelihood. Further, many have faced depletion in savings during these times, disrupting plans.

Stress and frustration over the drastic changes, social restrictions, and monetary issues have directly or indirectly influenced addictions [109]. According to the British Liver Trust, there has been a 500 percent increase in alcohol abuse calls for helplines [110]. Other parts of the world have also encountered similar situations. Workers who have been laid off and are jobless have resorted to substance use, drinking, and smoking. Furthermore, there has also been an increase in cases of domestic physical and mental abuse as abusers are at home for long hours and with the monetary issues, they may fault and abuse the victims further [111]. Russia also observed an increase in cases of drinking and a subsequent increase in domestic violence [112]. According to the Hindu, domestic complaints have witnessed a 10-year high due to the COVID-19 lockdown [113].

**Figure 6:**
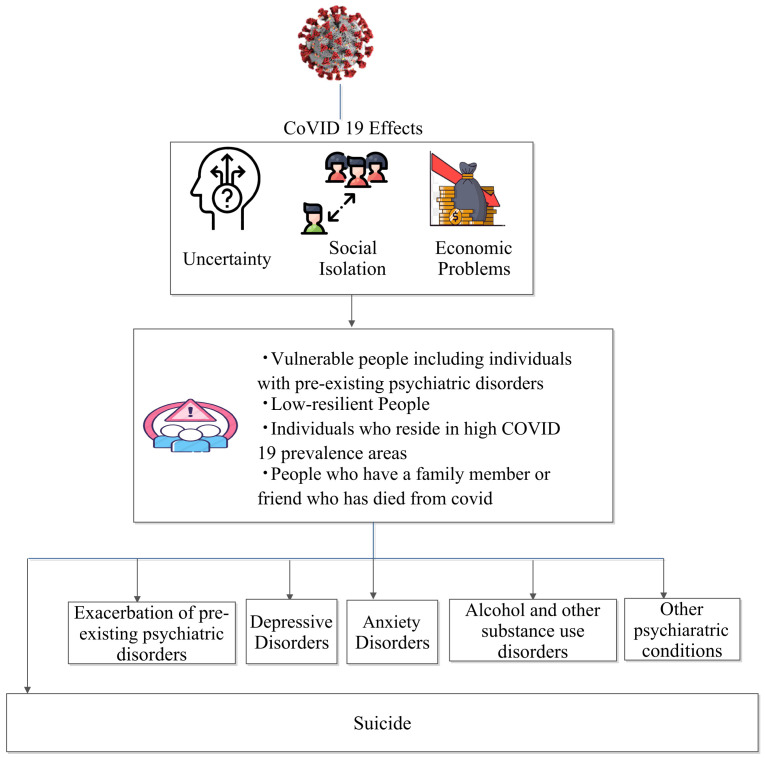
Psychological factors driving the increase in suicides due to COVID-19 [115].

Aside from drinking and domestic violence, there has also been an increase in the number of deaths by suicide. Figure 6 depicts a number of factors that contribute to psychological stress and mental imbalance, both of which enhance the suicidal risk as a result of the pandemic. The increase in cases of depression has also been a major factor. Furthermore, for academics, with the examination schedules unsettled, senior secondary school students and graduates have been under huge stress regarding college and job proceedings. This had been more so in India, where there was no surety regarding entrance exams for either. The online mode has also increased the already existing competitiveness even more. According to Medspace, in the U.S. alone, 53 percent of college students and 62 percent of high school students reported increased stress since the start of the pandemic, 48 percent and 51 percent experienced anxiety, and 33 percent and 38 percent suffered depression [114]. All of this has also contributed to the increase in cases of depression and suicide.

Thus, for the majority of the population around the globe, COVID-19 has deeply affected their mental state and psychology.

### 3.5. Behavioural

The behaviour of a person results from the mental state and situations experienced by that person. Several important theories such as the self-determination theory [116,117], throw light on the importance of social relatedness and psychological changes are key drivers of human behaviour.

Due to COVID-19, behavioural changes can be seen in terms of—changes in sleeping cycle, dietary behaviours, work attitude, buying habits, physical activities, behaviour with friends, colleagues, and family, personal-hygiene habits. Further, to cope up with the shift of old-school approaches into digital technologies, behavioural changes are trivial. There are theories such as Seligman’s PERMA model [118,119] and Diener’s tripartite [118,119], which identify the important elements of subjective well-being, suggests that subjective well-being is not the only factor that determines physical health; instead, it is the factor that determines overall health behaviours. With the help of such shreds of evidence, it can be inferred that behaviour is not independent of economic, social, health, and interpersonal relations of a human being. Anti-epidemic measures such as reduction in physical activities, social isolation have arbitrarily disrupted the subjective well-being index associated with behaviours.

Through study, it is observed that there is a significant change in individuals’ sleeping hours. This change divides people into two groups: first comprises people with a decrease in sleeping hours and second with an increase in sleeping hours. Sleep plays a pivotal role in the exacerbation of various kinds of illnesses. A recent study conducted at King’s College London claims that 38 percent of people have reduced sleeping hours or are deprived of good sleep [120]. As a result, 49 percent of people feel more anxious and depressed; this can be considered direct consequences of COVID-19 [120]. Sleeplessness leads to repercussions such as developing a high long-term risk of chronic diseases, worsening existing mental or physical health problems, and negative impacts on psychological state. A person with sleep loss can also have weak immunity; as a result, ends up suffering from various diseases, including COVID-19. Those who are experiencing poor sleep are more prone to alcohol consumption, believing that this will help improve sleep disorders. As mentioned under the psychological aspect as well, many countries recorded a rise in alcohol consumption. Some of the data shows a rise of 86 percent in Australia, followed by the United States and the United Kingdom with 27 percent and 22 percent, respectively [120]. There are other reasons for sleep loss, but there are other reasons for vulnerability towards alcohol consumption: boredom, inactivity, stress, anxiety, economic problems, and interpersonal relationship issues. The short-term effects of alcohol consumption are deterioration of the immune system, particularly affecting the lungs and liver, increased severity of existing diseases, a rise in recovery time, and significant elevation in domestic violence. Research studies claim that between 25 percent to 50 percent of domestic violence committers are drunk while attacking [120]. Some countries such as Lebanon reported almost double the cases of domestic abuse and France witnessed a 32 percent rise in such cases during the COVID-19 pandemic [120]. The other group with an increase in sleep hours as permitted by lockdown due to COVID-19 majorly consists of adolescents. Due to home-school, they can plan their schedule and have flexible sleeping hours resulting in a range of cognitive improvements, including an increase in the level of alertness, attention, memory, decision-making abilities, and problem-solving capabilities [120]. Thus, the long-term outcome of these cognitive improvements is academic enhancement.

Lockdown and restrictions on social interaction inevitably disrupt daily physical activities that balance physical and mental health. Many people find a negative impact of change in levels of physical activities such as going to the gym, walking in the park, and other recreational activities that improve a person’s mental state. Moreover, there are changes in dietary behaviours and the change in physical activities, which sum up together and leave adverse effects on an individual’s overall health. Due to the COVID-19 breakdown, the media reported that people are more prone to panic buying and stockpiling household items and as a result, they end up using more goods and preparing more food than normally required. Thus, through a study, Kantar Worldpanel believes that there is an increase of 38 percent of meal consumption during the lockdown [120].

Snacking habits are developed more with stress, anxiety, and boredom, and due to overeating, the problem of weight gain and obesity links up. On the other hand, a group of other extremes skip their meals due to stress, depressive and anxious feelings, and land up with issues such as weight loss and malnourishment [120]. An array of other chronic diseases such as coronary heart disease hyperlipidaemia, type 2 diabetes mellitus, stroke links up with changes in dietary habits [120]. Other factors such as the overuse of electronic devices and increased screen time exposure lead to tiredness and exhaustion, which promotes poor dietary habits.

There is another segment of behavioural changes that consist of changes towards other people and practices followed by public places. Most of the respondents refused to go to public places until and unless it is unavoidable. As per the survey conducted in China, almost all respondents avoided crowded public places to evade physical contact [121]. At least 70.9 percent chose to take preventive measures out of that, 98 percent chose to make fewer trips and avoid contact, 83.7 percent chose to wear masks, and 82.4 percent practice hand hygiene repeatedly [121]. The working class of society prefers to work from home rather than work daily to avoid physical contact and the risk of getting infected by the coronavirus. While meeting friends and family members, every individual tries to avoid handshakes and hugs in new normal conditions. Many people have developed obsessive compulsive disorder for personal hygiene due to fear of getting infected. As per new guidelines provided by the government, at every public place such as malls, shops, restaurants, banks, temples, and other corporate offices, it is compulsory to have sanitisers and wear masks at public places.

Thus, we can say there are drastic changes in human behaviours worldwide, ranging according to age, gender, regionality, and to which working class an individual belongs.

### 3.6. Interpersonal Relations

Interpersonal relationships are widely held to be an important determinant of human beings’ overall mental health and well-being. Various studies are held regarding mental illness, but it is important to study the core reason behind mental illness and also factors affecting mental health, which also play a role in understanding the aetiology of depression and other psychological distress up to a certain extent. It is equally important to study how each factor, such as socio-economic state, health, academic, behaviour, and the psychological state affecting the mental health of humans interact with each other. In the tough times of COVID-19, the whole equilibrium of these factors is adversely affected. Out of them, interpersonal relationships are affected to a greater extent. These studies adopted three kinds of approaches—by counting the number of social relations, detecting the quality and support of relationships, and societal predictors of suicide. After rigorous study, it is found that people who lack socio-economic support, mental support whose interpersonal relations are weak and are majorly vulnerable to depression, anxiety, suicidal thoughts, stress, all of these aggregates and directly affect physical health and indirectly lower down the immunity to fight against diseases. In this new normal interpersonal relations are affected at every stage of life—relations with family, friends, colleagues, people at the workspace, teachers, relatives, and workers.

Human interaction within private and public spaces took a whole new dimension as people spend most of their time being at and by isolating themselves to avoid unnecessary risks while meeting people. Stay-at-home, work-at-home orders have bound people and turned living spaces by spending the whole day at home in proximity to their family members and, in some cases, alone. Both cases have different kinds of effects—people who are alone in their homes feel loneliness, lack of physical interaction, lack of psycho-social support, which makes them cut themselves from society, lack of motivation to learn something new, and indirectly affects their work.

### 3.7. Academic

COVID-19 has created disruptions in educational systems. As per the United Nations, 1.6 billion of the learners are sufferers in more than 190 countries [122]. According to UNICEF, 46 nations implemented complete closure and 27 nations implemented partial closure that ended up affecting 72.9 percent of the world’s student population [122]. The majority of them belong to lower or lower-middle-income nations [123].

COVID-19 has widened the gap between higher-income countries and lower or lower-middle-income countries by exacerbating pre-existing disparities- fewer opportunities for learners from poor or rural areas, difficulties for a girl child to receive an education, and poor education facilities for students with disabilities [124]. Closures of educational institutions hamper the provision of essential services to children and communities, including access to nutritious food, affect the ability of many parents to work, increase risks of violence against women and girls, unequal access to educational resources, thus adversely affecting the socio-economic condition of the country. There is a high probability that learners with lower or lower-middle-class family background tend to drop out from any level for their education ranging from pre-primary to graduate studies and UNESCO estimates that 23.8 million additional children and youth may drop out or not have access to school next year due to the pandemic’s economic impact alone. School closures introduce other problems into society—child labour, child marriage, gender-based violence, early pregnancy, suicides—and hindered overall growth. The ripple effect of school closure also hampered the provision of essential health and nutrition services, which affected 370 million children in 195 countries, increasing hunger and nutritional deficiencies for the most disadvantaged [122].

**Figure 7:**
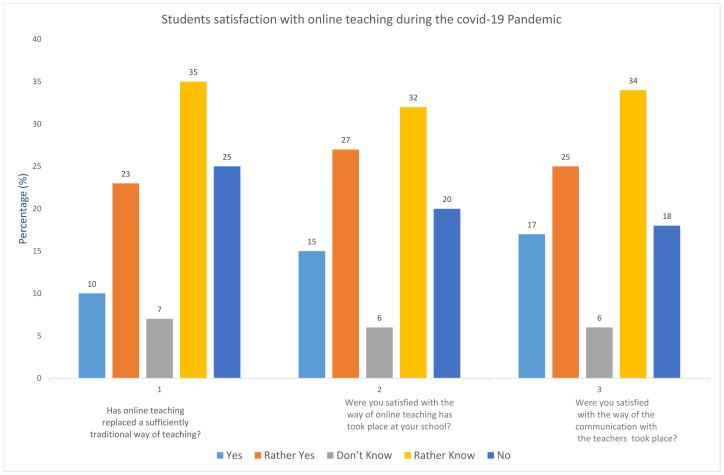
Student’s satisfaction with online teaching mode [127].

To ensure learning continuity during tough pandemic times, governments worldwide started imparting online lessons in urban areas using ICT tools, and for rural areas, more traditional methods mix educational television and radio programming, and the distribution of print materials are implemented. Distance learning in high-income countries covers about 80–85 percent, while this drops to less than 50 percent in low-income countries [122]. This gap is attributed to limited access to electrical services, weak digital infrastructure, low levels of digital literacy among students, teachers, and parents. People are trying to execute innovative and creative methods to spread knowledge and promote active learning is the positive side of the coin. Various techniques to monitor student’s progress with mobile phone surveys, tracking usage, and performance statistics from learning platforms and apps, are executed with rapid learning assessments to mitigate the learning gap. Some countries are developing educational materials and methods that can be accessed by disabled learners such as audio lectures, sign language narration, simplified literature; along with this, and there is a provision of assistive devices. Figure 7 shows a survey of students and their level of academic satisfaction after various online teaching capabilities that were provided to bridge the gap between traditional and online education. Usage of the transparent mask so that the deaf can lip read is an example of a simple, innovative idea that has achieved high effectiveness [125]. UNHCR has taken measures for children of rural areas to ensure access to distance learning by offering health facilities and sanitation facilities in and around learning spaces [126]. According to the United Nations, more than 70 countries adapted their school feeding programs to continue supporting children during school closures, about 50 arranged take-home rations to children and their families in various forms, including through daily meal delivery pre-packaged monthly rations. A total of 22 countries opted to replace the meals with vouchers or cash that families can use to buy food or other essential items. Some 6.9 million learners in 45 low-income countries have been reached since the onset of the crisis with take-home rations by governments [122].

The repercussions of the impact of COVID-19 on education include intense psychological issues such as stress, hypertension, depression, anxiety, panic attacks, insomnia, disruptive disorders, and neurocognitive disorders. The factors that can be taken into consideration are how prone a person is to mental stress/anxiety, how adversely academic performance is affected, technical issues, scarcity of required resources, reduction in income issues, how prone a person is to headache, earache, back pain, neck pain, or weak eyesight due to increase in usage of digital devices, and peer pressure.

There remains a downward cycle of progress, yet every downward cycle of academic circumstances there’s a reverse image of a positive cycle, which leads to the future of holistic development- inclusive of positive changes in the education system, radiating creativity and innovation of individuals, and achieving a mentally healthy and stable state for people.

## 4. Main Findings, Challenges, and Future Research Directions

Table 3 discusses the various technical solutions to mitigate the effects of COVID-19 on different aspects of human lives. These solutions are primarily from the domains of IoT, Blockchain, AI, and AR/VR, with one section of the table dedicated to the different technology-aided wearables that have been particularly useful during the pandemic.

IoT has helped with different provisioning aspects to facilitate data connection and networking via the Internet and provide the physical measurement to various parameters such as psychological health, changes in human emotions, frequencies of cases around an individual, amongst others, with real-time updates. Both software and hardware-based IoT applications mentioned in Table 3, such as smart thermometers, non-contact thermal scanners, and telemedicine, have proven instrumental in aiding the healthcare sector. Usage of IoT in drone-based delivery has helped the supply chain in benefiting the economic aspect. IoT applications have also been used towards psychological recognition through emotion detection.

Many wearables incorporate IoT and AI. The healthcare sector has also seen involvement from the AI domain for disease surveillance, risk prediction, and virus modelling and analysis. AI has also contributed to the psychological, behavioural, and economic aspects through the creation and launch of chatbots, telehealth checkup algorithms, psychological stressors, and other such AI-oriented services as shown in Table 3.

COVID-19 has brought in, along with these AI and IoT services, a large amount of unmonitored data. Through Blockchain, these data can be arranged into blockchain’s distributed networks to ease the monitoring while ensuring the required privacy and security. Table 3 shows we can set up different health records platforms, collaboration architectures, and donation or volunteer systems.

While the other aspects of life have majorly seen AI, IoT, and Blockchain-based technological solutions, AR/VR has played a large role in academics, with various conferencing platforms emerging as a key resource for online classes. Table 3 mentions the importance of AR/VR in distance learning and general higher education. At the same time, AR/VR also has involvement in the economic, behavioural, social, and psychological aspects with its usage in tele-immersion, surgical navigation, virtual tours of tourist spots, and many other such areas.

Other than the ones mentioned in Table 3, various domains such as 5G technologies have also played their part in this pandemic. AI, IoT, Blockchain, and AR/VR are discussed in further detail below, particularly their role in COVID-19.

### 4.1. Present Solutions and Challenges

#### 4.1.1. IoT (Internet of Things)

IoT has been widely explored and used by researchers to propose solutions for various problems faced by COVID-19. Some solutions have been checked through simulations, some implemented, while some have only been proposed as a probable answer. Some of the widely used devices were created years ago but became useful due to COVID-19. Herein, such different devices and technologies shall be explored.

The smart thermometer that has been deployed to major households in the United States was initially launched by Kinsa Health eight years ago to track common flu; however, it has been particularly useful in the pandemic since the thermometer is linked to a mobile application using IoT. Through this, the readings are forwarded to the company immediately. These data, once collected, are used by Kinsa to monitor different regions and notify the authorities of potential hotspots. Another similar application of IoT is the non-contact thermal scanner proposed by Bhowmik et al. [40]. In this, the Raspberry Pi 4 computer is connected to the GSM Sim for implementing timely alerts to the registered mobile number, to the ultrasonic sensor for sensing and alerting any cases of abnormal temperature, to an MLX90640 IR thermal sensor camera for operating it without contact and to a TFT display for displaying the detail. This hardware combination then forwards the data to two proposed applications using IoT where the doctors use one application and the other by patients. While the model proposed only provides non-contact thermal scanning and Global Positioning System (GPS) tracking to provide details of nearby hospitals, the future extension includes the provisioning of active GPS tracking to monitor patients closely and also work through contact tracing.

IoT Buttons named Wanda QuickTouch have been used by hospitals in Vancouver for rapid deployment in facilities requiring prompt sanitation or maintenance issue alerts to management. These are battery-operated IoT buttons that can stick to almost any surface. They would have been particularly useful in overworked medical units had their use be expanded to other regions.

Another application of IoT in COVID-19 is in the field of telemedicine that has seen a rapid rise due to COVID-19. In this, the patients are monitored remotely through various Internet of Medical Things (IoMT) platforms. In recent months, various telemedicine tools have been devised and used in different parts of the world. These telemedicine tools also include medical drones that have brought in increased use of another IoT feature—the Internet of Drones (IoD) [128]. IoD is used not only for medical purposes but also for general non-contact supply of goods.

While the above applications was healthcare oriented, one particular application of IoT focuses on the psychological and behavioural aspects wherein IoT-based emotion detection is extended to Long Short Term Memory (LSTM) based emotion recognition. While currently a proposed paradigm, it is expected to assist those in the academic field should any such outbreaks arise in the future. For future research directions, Awais et al. suggest focusing on end-to-end communications and visual aids for distance learning, alongside the incorporation of edge services.

The use of IoT also extends towards different types of wearable devices, which have provided various benefits towards mitigating COVID-19.

#### 4.1.2. Blockchain

Blockchain has been applied to most other domain applications—either for providing privacy, security, or ensuring high accuracy of information.

In assisting increased testing and reporting, blockchain technology can help ensure efficient and intelligent testing alongside data accuracy with regards to the number of tests performed. For this, distributed check-up points can be set up and corresponding nodes for a distributed blockchain network. Another instance where blockchain is of extensive use is secure and instant storage of COVID-19 patient records.

The blockchain network could also ensure effective implementation of lockdown through proper communication amongst authorities and efficient distribution and utilisation of services inaccessible and remote areas.

Further, using a public blockchain network in cases requiring high surveillance, such as during the pandemic, for information spread can allow the authorities to curb rumours or fake news. Blockchain-based incentive schemes such as the Reputation, Barter, and Credit Schemes implemented through the P2P distributed network can be used to set up an incentive-based volunteer participation system. Further, blockchain is helpful in setting up secure donation platforms worldwide to provide funds to the underprivileged.

Blockchain also provides security for the IoD venture and the sharing of health records among the medical staff. Applications such as Civitas and MiPasa, built for assisting authorities and sharing health records, also take help of the security and data assurance provided by the blockchain technology.

Since blockchain is a relatively unexplored field, the technology associated with it might encounter issues related to scalability and integration with legacy systems. Moreover, lack of awareness makes people believe that blockchain is only related to cryptocurrency and fraudulent activities. Next are scalability issues, as there are few platforms for blockchain and that have inherent constraints. As any central authority is absent in the blockchain, it is important to monitor such applications’ standards and designs.

#### 4.1.3. AI (Artificial Intelligence)

AI has proven to be a game-changing technical breakthrough since its inception [129]. It can be a highly useful weapon in the fight against the COVID-19 pandemic if used correctly. Any of the current and possible ways AI can assist authorities in successfully combating the COVID-19 pandemic are mentioned below.

The contribution of AI in healthcare, psychological, behavioural, and economic aspects of human life is very revolutionary. First, we cover healthcare and psychological aspects. In this, there is a successful Canadian-based AI model backed by machine learning and NLP-based algorithms BlueDot. This model predicts the spread of infection way before epidemiologists can do. A similar model was developed in Boston in 2019 named HealthMap [130]. Metabiota has created an infection surveillance tool that enables it to predict disease transmission. Metabiota bases its forecasts on clinical characteristics of the virus, fatality rates, and medication availability. The Epidemic Tracker from Metabiota also provides accurate details and up-to-date data on over 120 novel pathogens. The GVM (Global Virome Project) is an AI-based model that can predict the zoonotic viruses that can harm humans with the help of genetic and ecological databases of viruses in various animal species. These models were based on disease surveillance.

AI predictions of the distribution of COVID-19 are not yet very precise or consistent due to a lack of evidence, noisy social media, big data hubris, and algorithmic dynamics. As a result, most monitoring and forecasting models do not employ AI techniques. Most forecasters, on the other hand, favour well-established epidemiological models, referred to as SIR (Susceptible, Infected, Removed) models [130]. There are AI models that can calculate every individual’s vulnerability index based upon factors such as age, travel history, hygiene habits, current health status, pre-existing health conditions, and family medical history that show how susceptible a person is to COVID-19. For risk predictions, we use face scanners, AI-based CT scans and x-rays, and intelligent voice detecting systems. Tableau has developed a COVID-19 Data Hub with a Python script to show how to retrieve data from the New York Times’ COVID-19 dataset and generate data visualisations of the infection’s progression [130]. It is critical to consider how this virus spreads and which hosts are responsible for its dissemination. As a result, scientists suggested AI and machine learning models for detecting hosts using databases of documented identical viral genomes. There are models that are emotionally intelligent which use AI and NLP, which are used for teletherapy. Then comes chatbots into the picture, which serves a wide array of solutions for marketing, customer service, mental support, and provides psychological help.

**Figure 8:**
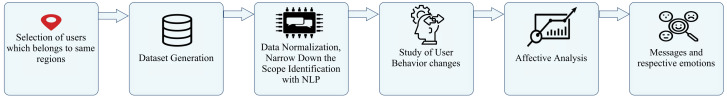
General procedure for functioning of chatbots.

Figure 8 depicts the generic steps involved at various phases of the chatbot. When a user attempts to communicate with a chatbot, a database record is established based on their location, which is then narrowed down for scope identification using various NLP algorithms, followed by the detection of behavioural changes and effective analysis. Following these processes in a real-time context, replies with corresponding emotions are formed. Health chatbots can ask questions, create health records, generate reports, and have conversations [56]. The event detection system consists of five modules, each of which incorporates various AI sub-components for changing user behaviour in online social networks [53]. Topic recognition and affective analysis were needed and an interpretation of the event’s main characteristics, the methodology behind that is explained. The epidemic, now pandemic, and its aftermath became a fertile ground for false news, affecting human behaviour, mental health, and the human environment as a whole. Big corporations, including Google, YouTube, and Facebook, are using artificial intelligence (AI) to spot false news, and they were able to halt the spread of rumours, conspiracies, and disinformation on large social networking sites up to a certain point [130].

There are many challenges and limitations for AI-based models and technologies such as the first and the biggest issues are lack of data for training and testing models, as this is a novel situation. If data are available, then it is noisy or full of outliers. Secondly, these models breach public privacy as they use important sensitive data. Another drawback is that human intelligence is very important while designing models, handling pre-processing and training data, and analysing results to conclude.

#### 4.1.4. AR (Augmented Reality)/VR (Virtual Reality)

AR (Augmented Reality) and VR (Virtual Reality) have been used for years and new technologies are emerging through various researches and studies which include MR (Mixed Reality) and XR (Extended Reality). Devices backed by these technologies have been used for ages, and new emerging solutions are used to ease COVID-19 tough situations. Here in this section, we explore such devices and technologies and we also see how it provides solutions for different parameters of human lives.

In the healthcare sector, AR/VR solutions such as customised surgical navigation and provision of hardware for diagnostic IoMT [65] are used in pandemic situations. Customised surgical navigation is performed by combining AR, neural networks, and haptic rendering-enabled surgical tools [64]. The combination is used to provide a novel technique for image-guided COVID-19 lung biopsy with accurate results. On the AR side, a haptic—an AR guiding strategy is introduced that accounts for precision and reliable surgery [64]. A 3DUI, including all requisite surgical details, was devised with a real-time response guaranteed. AR/VR lead to minimal invasive surgical image-guided surgery (IGS), with light injuries, traumas, and minimum recovery time, bringing a revolution in the field of biopsy and complicated interventional surgeries. To achieve a better human ergonomics performance, visualisation is performed using all the navigational clues from the Haptic-AR guide system [64]. Diagnostic IoMT solution for the COVID-19 telemedicine diagnostic combines customised 5G technology, deep learning algorithms, and extended reality [65,131]. The role of extended reality is towards provisioning of remote surgical plans and rehearsing hardware to create a surgical decision system.

As a part of economic sector solutions, we throw light on factory automation, virtual tours, setting up of workspace in work from home situations [66]. Regions whose primary source of income is tourism face a lot of difficulties in a pandemic situation, so to solve this, AR/VR-based virtual tourism was started and people were earning income through this technology. Using the AR/VR and high-quality streaming and holograms alongside the required 5G technologies, e-tourism solutions can be developed [69]. Moreover, it could help mitigate the sense of isolation and depression felt due to the restricted movement resulting from the pandemic. To do complex assembly remotely, AR is used, which makes instruction manual and complex drawings visible via AR glasses. It is used for manufacturing smartphones to jet planes and anything complex. AR-based solutions such as remote maintenance solutions contribute to more effective automation due to the reduction in on-site workers in COVID-19 breakout. This may help factories in functioning to their best capacity, which in return improves the economic state. VR is used to educate workers in fields such as manufacturing, design, real estate, and those that include human contact with real-world objects [69]. It is used in the medical field to diagnose and treat patients who have visual or cognitive impairments. Welders, plumbers, engineers, oil workers, and those who need to be hands-free when working in factories and fields use AR headsets [69]. Customers benefit from an improved shopping experience thanks to AR/VR, and they can upgrade their homes remotely by interacting online with an interior designer or architect [69]. VR-based applications that have a massive effect on a person’s productivity from home have been proposed to acquire the benefits of a physical workspace in work from home scenario.

The use of audiovisual reality, as well as SDN and NVP 5G technologies [58,59] to provide proper connectivity, is used in distance learning, which is real-time online education. Experiential and interactive learning are very common in higher education, and certain ideas cannot be learned without simulating them, so AR/VR applications are extremely important. VR creates environments that are not readily available to students studying space science, archaeological studies, chemical engineering, medical science, aviation-related studies [59,60,61], etc. This is also used for military training. Tele-immersion [71,72] is a new concept that enables people to co-exist in the same room while communicating realistically physically. This plan could help friends and families digitally bond in the circumstances such as mutual distancing, reducing social anxiety and loneliness.

**Figure 9:**
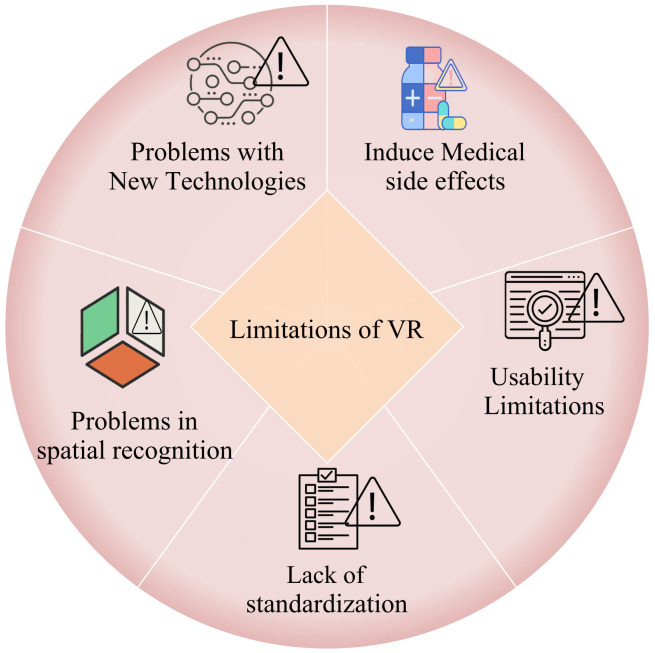
Top limitations of VR [133].

There are certain AR and VR technology limitations out of them some top limitations are mentioned in Figure 9. Apart from the others comprises of the cost of devices and equipment are very high. The software for virtual reality is considerably larger than the hardware for virtual reality. It is a given that VR systems need more programming to have an immersive environment. Compared to other computers, VR apps take up a lot of space and need a lot of processing resources. These devices are likely to cause locomotion sickness and can also damage eyesight if it is exposed for a longer time [132]. The problem of privacy is a major source of controversy when it comes to technology. Users may view information about strangers from their web accounts using image recognition tools and AR.

#### 4.1.5. Others

Other than these four major fields, 5G technologies also play an important role. The majority of the applications mentioned above incorporate these 5G technologies at one phase or another. Particularly, wearable devices that use AR or IoT also perform more efficiently when connected via 5G technologies.

### 4.2. Future Research Directions

Motivated by our detailed survey on research studies, we point out possible research directions that should be considered in future works.

#### 4.2.1. IoT

IoT has been an integral part of technological implementations towards mitigating COVID-19 and the pandemic; however, there have been a few issues that have particularly come to light in the wake of COVID-19. These include scalability issues, limited spectrum and bandwidth, security and privacy issues, and big data centres’ requirements, amongst others [134].

Implementing IoT for wearables and IoMT services has seen the use of a large number of IoT hardware, such as sensors, since a single IoT gadget can require multiple sensors. This, along with the other matter about the large amount of data floating around the IoT nodes, increases scalability issues and energy requirements [134].

Further, currently, most of the IoT devices have a limited available bandwidth based on the licensed spectrum that they use. Now, with the growth in these devices, bandwidth issues occur, and the data faces latency, which may introduce an error into the data transfer [134]. In hard real-time situations such as the COVID-19 emergencies, timely and correct data transfer is of utmost importance.

Moreover, the growing data have caused big data storage centres to store the data.

To mitigate some of these issues, certain considerations for future aspects are to be contemplated. Scalability issues could be solved by introducing lightweight security algorithms based on the different metrics, including the angle of arrival, time of arrival, phasor information, and received signal strand indicators.

Another method for the same could be to bring in more and more software-defined IoT to minimise the scalability issues and attain a logically centralised control for data collection and monitoring [135].

Further, more and more efforts towards strengthening the security are looking towards energy-efficient primitives that take up less memory with less computational complexities [135].

The limited bandwidth issue can be avoided via the proposed cognitive-radio-enabled IoT [134,136]. Through the cognitive radio parameters, the devices can sense the environment and detect the unused frequencies on their own and then select the most appropriate spectrum based on their quality-of-service requirements [134].

#### 4.2.2. Blockchain

Blockchain has been essential in various domains during COVID-19 ranging from COVID-19, disaster relief and insurance, and patient information sharing to agriculture, supply chain management, and e-governance. This diverse use has brought certain challenges to the forefront [137].

Blockchain suffers from the lack of a skilled workforce that specialises in the domain. Blockchain platforms require security, app development, and engineering skills, amongst others. Since there are not many people who are well versed in all of these fields, few people explore blockchain further.

So, with the requirement of blockchain-oriented technologies increasing due to COVID-19, a high shortage of skilled employees has been seen in companies, and it is particularly problematic for them, in the pandemic scenario, to nurture new hires regarding the same. They have started in-house training and outsourcing to compensate for the same [138].

The blockchain network involves distributed databases that create decentralised automation. Further, along with decentralisation comes anonymity. This clashes with the existing legal norms that require someone to hold authority in case of any disputes [139].

Latency, throughput, and scalability are also some of the major challenges for blockchain. COVID-19 requires real-time functioning and response; however, with the bulky network traffic that blockchain experiences, the latency might range up to a couple of minutes with the current conditions. This can then result in lower output and hence affect the scalability [138,139]. Certain solutions have been proposed to mitigate the same. These include the novel scheme proposed by VerSum [138,140], sharding [139,141], and layer—there are two scalability solutions [139,142], amongst others.

Privacy issues are a major concern for blockchain networks. Since the blockchain nodes can access the entire database, it gives rise to privacy issues for an organisation’s confidential data or the user’s data. Moreover, different data processing and storage methods at different stages further increase the vulnerability of private data. One method to ensure the privacy of the user and the organisation is to use distributed off-chain storage [139] to make the data available as well as private. Along with this, new privacy methods, such as privacy-by-design and mixing, attribute-based encryption, and zero-knowledge proof, amongst others, can be used to add another layer of protection [139].

While blockchain is majorly secure, and it can still be vulnerable to certain attacks mounted onto the blockchain application. Be it wallet-hijacking or crypto stealing malware, these attacks do not have to deal with the secure blockchain infrastructure and can be easily deployed onto the application [139]. Further, with the advancement in quantum computing, attacks may become more frequent in the future. For this reason, better encryption techniques have been and are still being designed [139].

#### 4.2.3. Artificial Intelligence

As we saw in the earlier sections, we used AI to fight against COVID-19; however, we came across many challenges present in existing solutions, which include lack of data, noisy data with outliers, big data hubris, unorganised data, and algorithmic dynamics [143]. Due to inconsistent and unreliable data, it is not easy to train AI-based models to predict and diagnose coronavirus.

The issue of insufficient data can be solved by incorporating open-source data platforms. The reproducibility, openness, open science, and open research help us create a public trust for open data, which can be used to train models [144]. The pursuit of scientific research in a collaborative and barrier-free environment streamlines knowledge development, fosters discovery through unrestricted collaboration, and improves the robustness of findings through inclusive evaluation and peer review [145]. Open-source data-sharing initiatives taken are the World Health Organization provides coronavirus disease datasets and the Allen Institute of AI, Microsoft, and Facebook, provides 44,000 open-source articles that can be used for data mining. Similarly, Elsevier, ScienceDirect, The Lens, Google, and Amazon provides data pools that are curated and up-to-date [130]. This solves the lack of data, but again too much data can introduce redundancy and big data noise, resulting in overfitting the model [145]. This can be solved by content curation and algorithmic changes, which are performed manually by human beings but can be performed automatically by incorporating machine learning and natural language processing. The next issue of noisy data can be solved by thorough data preprocessing and various data mining techniques applied on huge datasets before using it for models. More sophisticated and effective ways of data mining techniques need to be found to solve data-related problems. There are issues related to inconsistent and unorganised data, which results in false predictions. These problems can be data labelling, data partitioning and data organising from multiple online resources. There exists a lot of data in multiple languages and multiple forms, so we need to convert them into one standard language and form so that any AI-based model predictions can be applied globally [146]. Other miscellaneous issues are either due to human error, faulty readings from sensors, fake data in the global database, and likewise many more. For example, COVID-19 detection cannot be performed properly using thermal sensors in cameras because it cannot obtain accurate readings of people wearing spectacles [143]. Further, the resolution of images used for the detection of COVID can create problems. Transfer learning is used to obtain advantages but over-parameterisation of models hinders performance, so there is a requirement for more accurate image processing techniques [145]. Every useful model for diagnosis or prognosis must produce accurate results for every sample from the target population, not just the sampled population [145]. More human intervention is still required to apply and understand data before predictions are made using AI models; therefore, we require more automated solutions to reduce human errors. So there are certain open challenges present in existing AI-based solutions, which can be solved by further research.

#### 4.2.4. AR/VR

We have seen many applications of AR/VR in previous sections that are used for combating COVID situations, but that comes with certain limitations and challenges that are yet to be resolved. Challenges such as security, data privacy, health issues, content quality, health-related issues, data streaming, lack of awareness, and other social issues.

Lack of design standards results in models that are not compatible globally and it will make overall development slower [147]. AR/VR technologies obtain access to sensor data, video and audio of devices, GPS, etc., so any malicious attack or breaching of data are a very high risk. Breach notifications are expensive and time-consuming [148]. To ensure the privacy of data edge devices without sending data directly to the cloud server. To improve the spatial recognition capabilities of our platform, we can use a variety of sensors to obtain COVID-related information at different levels. Adding to the spatial database by aggregating all information about individuals in the room, as well as humans’ biological information in the environment such as heart sounds, nausea, and headache that cannot be seen by human eyes. This suggests that applying cutting-edge sensor technology would open up new possibilities for applications and research: more stringent privacy protections and considerations. Due to the absence of privacy and security protocols, there are chances that data can be manipulated easily and presented; this situation becomes worse because developers are reluctant to take appropriate actions before such things take place [147]. As AR/VR is incorporated into sensitive industries for operating machines remotely, for factory automation and in the medical field for tele-immersion and surgical navigation; it is very important to have high-quality content availability. Presently 5G technologies are used for the same, but this area requires further development to obtain higher precisions. There are hardware-oriented limitations that affect user experiences such as lack of precision, navigational issues, GPS errors, latency, and battery life [149]. To incorporate customisability and applicability to virtual models, native gestures and native NLP support on HoloLens are used; still, this requires further evaluation and research [150]. Hardware is quite expensive and inaccessible to all; also, their maintenance requires specialist knowledge.

Apart from these, there are health-related issues such as experience disorientation or a type of motion sickness known as simulator sickness. When developing applications for VR systems, developers should use techniques and best practices to reduce simulator sickness by better addressing problems such as delay, flicker, and acceleration, which have been described as possible contributors to simulator sickness [150].

## 5. Discussion

This review article aims to provide a comprehensive review of the technological solutions towards analysing the effects of COVID-19 and the pandemic on different aspects of human lives. The motivation behind this paper was the lack of any such comprehensive compilation that focused on multiple different aspects of human lives affected by the pandemic, and at the same time, looked into the available technological solutions pertaining to these aspects. Existing articles such as [3,5] focus on individual aspects such as academic and economic aspects, respectively. Literature such as [1] provides a regional analysis into the effect of COVID-19 on interpersonal relationships. There is no literature that covers the impact globally. On the other hand, Ref. [2] focuses more on the technological aspects whilst briefly focusing on some of the parameters such as the economy and healthcare-based aspects. Further, whilst [2] talks about the different technological advancements, it does not provide clear details about which human life aspect it aids. So, this review article can reduce this gap in the existing literature.

We have gained profound insight into how deeply COVID-19 affected human lives through this review paper. Be it the social, economic, interpersonal, psychological, behavioural, academic, or healthcare-based aspects, all these aspects have seen a sudden and huge change, resulting in adverse impacts in many cases. Social lives have taken a hit and people have further turned dependent on phones. Economies of countries and individuals suffered and while they slowly picked up the pace, they have not been completely revived due to COVID-19 and its variants. Interpersonal interactions between humans have reduced in-person and turned virtual. The pandemic has also affected people psychologically due to the sudden change. Students of various age groups, fields of study, and regions have unexpectedly adapted to a different teaching system. Whilst some have better adapted to this form of teaching, some have struggled. Healthcare suffered intensely. COVID patients have had to deal with the stress of quarantine and other possible health issues occurring simultaneously. Patients suffering from other diseases have faced neglect in healthcare due to the lack of facilities or fear of contracting COVID.

Despite all this, there have been some small positives observed in the pandemic, such as the slight improvement in the environmental conditions. Many families have been able to spend time together in a better way. Many companies have adapted to a hybrid model of office–working from home, which provides employees the opportunity to choose the best feasible option; however, people have experienced more suffering than gain in the pandemic. The technological advancements have helped mitigate COVID, the change, or at least providing a method to cope with the unforeseen change. Through AI, blockchain, AR/VR, and IoT, testing and healthcare provisions became easier, academics have improved, and data made more secure through the different technological domains.

## 6. Conclusions

To conclude, we have observed the different aspects of human life affected by COVID-19 and the resultant implications. We have then explored the different existing and evolving technologies that have been essential in aiding these different aspects. This paper throws light on the systematic categorisation of all the aspects of human life in COVID-19 and how to handle each parameter of mental aspect using various technologies. Considering how human life has been affected brings in different real-life problems that can be worked upon through technology integration. Major technical domains such as AI, blockchain, AR/VR and IoT have been incredibly helpful. They shall continue to be fundamental to developing tools and technologies that aid the social, healthcare-based, economic, psychological, behavioural, interpersonal, and academic aspects of human life. The balance between these aspects that has been disturbed due to COVID-19 can be restored to a certain extent with the present technology mentioned and can achieve even better results in the upcoming years. The advancements in technology resulting from the COVID-19 pandemic have provided a base for better-equipped facilities and preparation, should another pandemic occur in the near future or otherwise at a larger scale. It is expected that these technologies will act as a backbone for various kinds of therapies targeting the different aspects of human life. We believe our timely study will shed valuable light on the comprehensive research of the effects of COVID-19 on human lives, aid with technological solutions, and motivate the interested researchers and practitioners to put more research efforts into this promising area.

## Figures and Tables

**Table 1:** Identified research questions and their objectives.

Q. No.	Identified Research Questions	Objectives
RQ.1.	What are the different aspects of human life affected by the pandemic?	This question aims to explore the different aspects of human life that have been impacted by the COVID-19 pandemic.
RQ.2.	How has the pandemic affected these aspects?	It aims to search and tackle as to how the pandemic has affected the different aspects of human life.
RQ.3.	What is the existing literature that provides insight into the effect of COVID-19 on human lives and which aspects does the existing literature explore?	It aims to recognise the existing articles that are relevant to this research question and provide a detailed comparison between them to grasp the missing objectives.
RQ.4.	What are the different technologies that have helped in mitigating the adverse impacts of COVID-19?	This question aims to bring to light the usefulness of certain technological advancements towards mitigating COVID-19.
RQ.5.	How have the different technological advancements helped, and which aspects of human life have they aided?	It aims to provide the detailed review of the advancements and their effective role in aiding COVID-19 mitigation for the different human life aspects.

**Table 2:** Quality hiding questions.

Description of Question	Answer
Is this a broad examination of the effects of the pandemic on all elements of individuals ’ lives and the technological solutions in such a challenging situation?	Yes.
Are papers only related to COVID-19 and technologies (such as AI, ML, AR/VR, IoT Blockchain) are considered and others are discarded?	No.
Have the abstract, title, and full text of the research paper describes the taxonomy of parameters related to human lives that are affected due to pandemic and technological solutions?	Yes.
Do the abstract, title, and full text of research paper only describe the ‘‘health related pandemic impacts and technology in use?”	No.

**Table 3:** Relative comparison of the existing surveys with the proposed survey.

Related Surveys	Year	Objective	Key Contributions	Limitations and Open Issues
[1]	2020	Impact of COVID-19 and Quarantine on Interpersonal Relationships and Psychological Distress.	Provides a study-based analysis regarding the improvement or decline of different interpersonal relationships and also regarding psychological distress amongst the participants of the study.	The study is majorly restricted to people of Chinese origin and hence can not substitute a worldwide scale.
[2]	2020	Comprehensive Review about COVID-19 and role of different technologies in managing COVID-19.	Detailed review with information about COVID-19, other pandemics, impact on the global economy and different industries, diagnostic testing, and role of technologies towards aiding the testing and treatment.	While it provides details about the effect of COVID-19 on different industries, there are few details regarding the people employed in the industry and the technologies involved mainly focus on healthcare-based solutions and not the economy or other aspects.
[3]	2020	Effect of COVID-19 on digital education and the factors behind the same.	Tabularised statistical modelling approach stating all the factors alongside the tests.	The authors could not conduct a CFA-based factor analysis due to lack of existing literature to be taken as a base.
[4]	2020	Technical and human-driven approaches to control transmission.	Analysis of technical and human-driven approaches for indication of virus and mass quarantine, further details about interpersonal impacts in case of human-driven techniques, majorly.	The article only focuses on the two approaches to control transmission, unlike the various available solutions to fighting pandemics. Moreover, the article focuses on the differences in China and Western governments mid-way, with few details about the human-technology relationship. Further, as mentioned in the article itself, generalising the given information without caution can be an issue.
[5]	2020	Socio-economic impact of COVID-19.	Survey provides comprehensive details regarding the effect of COVID-19 on various economic sectors.	The survey does not give sufficient information regarding the social aspect and focuses majorly on the economic aspect only. Further, the survey focuses on organisations in the different sectors and not on the employees in that sector.
[6]	2020	Impact of COVID-19 on the global economy, and the global spillover.	Analysis of why COVID-19 had such a huge impact on the global economy and how COVID-19 has impacted the global economy and detailed review about the different policy measures in the fast policy response.	The analysis missed out on a few major industries impacted by the COVID-19. Overall, many different countries were included in the individual sector and the focus was on specific countries.
[7]	2020	Effect of COVID-19 on human psychology, the world economy and the environment.	The positive changes of COVID-19 towards the environment summarised well.	The world figures are superficial and do not give any detailed information regarding the economy, and the information regarding the psychological impact is generalised without proper context provision.
[8]	2020	Impact of COVID-19 on psychosis.	Review contained various details about a possible increase in psychosis due to COVID-19 and evidence for the same, also mentioned about adherence with protective measures and risk perception.	The review, as mentioned in the article itself, had traded time for data assurance, so only one of the authors had a hand in data extraction. Further, there was little mention of the effect COVID-19 had on people already diagnosed with psychosis.
[9]	2020	Psychological impact of COVID-19.	Detailed impact of COVID-19 on various psychological factors given along with risk factors, protective factors, and preventive strategies.	Data were taken from the early stages of COVID-19 and can not essentially be considered globally. Further, data were taken voluntarily, so there was no accountability.
[10]	2020	Behavioural Analysis of COVID-19.	Analysis about behavioural biases amidst COVID-19, majorly panic buying; various behavioural theories used to analyse the same.	Despite there being different behavioural theories, all of them eventually focused on panic buying instead of other human behaviours in such situations.
[11]	2021	Psychological impact of COVID-19 alongside restrictive measures on a global scale.	Psychological effect of COVID-19 considered through multiple variables and each of them measured through a survey, to obtain conclusive results about the impact.	Otherwise a well-constructed article, the survey being of online mode could result in the possibility of ambiguity.
[12]	2021	Addressing the impact of COVID-19 on mental health.	Detailed analysis about the various contributors to negative mental health during such situations such as the pandemic, possible primary and secondary preventive interventions.	The article takes on a population-based perspective and hence, less specified mental health impacts are missed out on.
[13]	2021	Impact of COVID-19 on the environment and the different aspects of society.	Provides detailed analysis on how COVID-19 has affected different parts of the environment, family life, society in general, employment, and education.	While there is mention of improvement in family life, there is no mention of the increased dysfunctionality in families due to being home. Other social aspects of an individual’s life are not mentioned.
Proposed survey	2021	A comprehensive review of the technological solutions to analyse the effects of pandemic outbreak on human lives	Provides detailed horizontal taxonomy of impacts of COVID-19 on human lives and their technological solutions,	—

**Table 4:** Main effected areas of human lives.

Parameters	Brief Description	Relevance to COVID-19
Social	Herein, the social aspects of human lives are talked about—social events such as weddings and family gatherings stopped. At the same time, the daily interactions with correspondents, the workspace environment, the air of familiarity around people sharing a commute, amongst others, also stopped or were restricted to a certain limit.	With social distancing a norm since COVID-19, an individual’s social life fell to shambles, in-person interactions turning virtual. Moreover, most countries in the world were observing lockdown as a mitigation measure for the pandemic. So, the daily social interactions were also reduced to a minimum. Hence, it is important to study how this change affected human lives.
Healthcare	Healthcare is always a sensitive aspect of human life since being healthy is an essential part of the human lifestyle. In the healthcare aspect, different age groups witnessed different effects.	While considering the effect of COVID-19, considering how the disease affected the patients’ health is of obvious relevance. Further, due to COVID-19, patients suffering from other diseases were indirectly affected; hence, this aspect of human life is also significant.
Economic	An individual’s life decisions revolve around the monetary funding they have. The current economic scenario also determines their future. Further, so is the case for a nation’s economy.	A pandemic such as COVID-19 shall affect most people’s economic situations and change the overall economy of nations. Hence, it is essential to study the effect of COVID-19 on the overall economic situation worldwide.
Psychological	Human psychology is inherently connected to other aspects of human lives and changes in these aspects, in turn, affect the human mind. Human psychology is constantly interlinked with an overall human livelihood, a driving factor behind the human response to situations.	COVID-19 has brought out many changes in other aspects of human life and these changes are ought to reflect in terms of psychology as well. The psychological effects of COVID-19 can have large and lasting consequences. Hence, it is necessary to study the mental effects of COVID-19 on human life.
Behavioural	Behavioural aspects of humans comprise every action any individual can perform, such as changes in sleeping cycle, dietary behaviours, work attitude, buying habits, physical activities, behaviour with friends, colleagues, and family, personal-hygiene habits. It is the reflection of the mental state of a human being.	A wide array of changes can be seen in the behaviour of any individual as after-effects of the COVID-19 outbreak. Due to stress, anxiety, depression, and the sleeping cycle, drug consumption has increased, and the suicide rate has increased. Due to the spread of fake news, people started hoarding things unnecessarily. Nowadays, every individual thinks twice before going to public places. There are many such changes. Thus it is important perimeter to study the effects of the COVID-19 pandemic on humans.
Interpersonal	Interpersonal relations are the building blocks of society. As human beings are social animals, it is important to understand how an individual thinks and acts with other people by whom they are surrounded.	Due to measures such as lockdown, social distancing and work from home taken to stop or reduce further spread of COVID made people feel loneliness, lack of physical interaction, and lack of psycho-social support, which makes them cut themselves from society. These all make them feel demotivated, which indirectly affects other aspects of any individual’s life. Thus, it is crucial to understand this perimeter.
Academic	Academic consists of an overall learning process and growing through that. Academic aspects of every individual irrespective of their age in studied under this as these people will contribute to society.	Academic system, which was earlier in-person, is now shifted to online virtual mode due to the COVID-19 outbreak. This shift brought a drastic change in teaching style, contents delivered and way of delivery. This factor is very important as it reflects the mental state of every individual who is directly or indirectly part of the educational system around the world.

**Table 5:** Present technological solutions to mitigate negative impacts of COVID-19 on human lives.

Technology	Solution	Description	Applications Targeted
IoT	Smart Thermometer	These thermometers are connected to mobile devices and in case of an increase in body temperature, the readings are shared to the related authority. This authority obtains real-time information of COVID-19 hot spots if many people in a particular area show high-temperature symptom.	Healthcare
	IoT Buttons	To reduce hospital-based infections and can send quick alerts related to public safety risks. The benefit of this button is it can stick to any surface and it is battery operated.	Healthcare, Psychological
	Telemedicine	Telemedicine carts, teleconsultation software, and portable tablets connected to smart wearables, mobiles, and other devices provide remote medical facilities to evaluate, diagnose, monitor, and cure patients’ disease.	Healthcare
	Non-Contact Thermal Scanning	Raspberry Pi 4, alongside a non-contact MLX90640 thermal camera sensor, screens people from a distance to determine whether they are affected. These data are stored in the cloud database and can be accessed by assigned personnel from any handheld device. GPS is used to track the position of patients as well as track the areas visited by the patient [40].	Healthcare
	LSTM-based Emotion Detection and Recognition	It involves an AI-based data processing system and an IoT-based data communication framework that analyses sensing modalities to interpret the inner inaccessible human system of emotions [41].	Psychological, Behavioral
	Layered Model for Predicting and Preventing Cyber Attacks	The IoT layered model contains three layers—end-user, sensor (device), and cloud layers. The stretched model two additional layers of edge computing and fog computing [42]. It was proposed that the increased online social platforms and the involvement of online meetings and cloud platforms due to the work from home scenario [43].	Social, Economic
	Internet of Drones (IoD) Architecture	IoD architecture has been set up to provide AI-enabled IoT based drone—aided healthcare services [44].	Healthcare
	Smart Speakers	While smart speakers existed well before COVID-19 and their usage saw a minor increase during the lockdown, the way they were used certainly changed. More users started using them for news and music. Further, adding a new feature to the speakers that could detect a lockdown outbreak was proposed. Participants of the questionnaire conducted by Furini et al. agreed that in critical conditions such as the pandemic, such a surveillance tool might be helpful [45].	Behavioural
Wearables	WHOOP Strap 3.0	This is a wrist wearable similar to Fitbit and other smartwatches, but its accuracy in measuring cardio-respiratory parameters is very high. This detects abnormal respiratory behaviour, which is a symptom in COVID-19 patients. It provides early warning for COVID-19 breakout in a particular region by collecting information globally. It is connected to smartphones, which help in monitoring behavioural aspect of the person.	Healthcare, Behavioural
	Estimate Workplace Level Contact Tracing Wearable	This wearable device is developed to enable contact tracing in a workspace. This device monitors the health of employees continuously and in case of any abnormal symptoms, it records it into a main central database. Bluetooth Low Energy (BLE) based proximity sensors keep track of contact of a particular employee in the organisation. This reduces the high risk of the rampant spread of COVID inside the organisation. Hence this provides a safer workspace.	Healthcare, Interpersonal, Psychological
	LifeSignals Biosensor PatchL	A wearable patch is designed that is kept on the chest area and will monitor ECG trace, heart rate, and respiration rate in real-time and which is connected to the user’s smartphone. If a person develops COVID-19 symptoms, then his/her data are sent to a healthcare worker to obtain better help.	Healthcare
	Spry Health’s Loop Signal	Loop Signal tracks the heart rate, respiratory rate, and pulse-oximetry of the patient, and collecting it through hundreds of data points in patient to obtain the utmost accuracy in analysing a person’s condition.	Healthcare
	SPHCC With Cassia and VivaLNK (Bluetooth IoT gateway + Wearable sensors)	Wearable sensors keeps on checking patient’s temperature in real-time, which is sent to healthcare workers who can remotely keep track of patient’s condition.	Healthcare
	Digital PPE	Open source low-cost wearables functioning using BLE, generally available as smartwatches, have been proposed by Woodward et al. for contact tracing and social distancing reminders [46].	Healthcare
	AR Glasses	These AR wearers help with social distance measurements, fever detection, and touch object detection, which could help in better analysis and mitigation of the COVID-19 scenario, ensuring better social distancing and at the same time, keeping the data regarding the same up to date [47,48].	Healthcare, Social
Blockchain	Civitas	A mobile app based on blockchain that monitors a person’s health and notifies the person whether he/she is permitted to leave home or not. It also provides features such as the ideal time to go to public places such as the market, which minimises the risk of spreading COVID-19. App also ensures that the sensitive private data of each user remain secure [49].	Healthcare
	MiPasa	This is an IBM blockchain and IBM cloud-based data streaming platform that shares the verified locations among individuals, healthcare workers, hospitals, and other relevant organisations. These data are crucial for hospitals prepared beforehand to deal with the COVID outbreak.	Healthcare, Economic (for hospitals)
Blockchain	Facilitating Increased Testing and Reporting	It is important to increase the number of COVID tests and accurately maintain that data where blockchain comes into the picture. There are multiple test centres distributed in the same blockchain network. These test centres act as nodes in the system and continuously updates the data in real-time. This network acts as a single source for healthcare officials, hospital, and individuals to retrieve necessary data. As IoT architecture is immutable, data stored in this network are completely reliable.	Healthcare
	Managing the Lockdown Implementation	Through blockchain distributed network governmental and non-governmental organisations can keep track of the needs of residents in a particular area and as a result, this increases the efficiency of frontline workers and brings balance in the supply of services.	Healthcare, Economic (reduce the waste of unnecessary funds by increasing efficiency and balance of the system)
	Preventing the Circulation of Fake News	By using a public blockchain network and consensus-based algorithms, the system can curb the flow of rumours, conspiracy theories, fake news, and inflammatory remarks. Further, in case if such fake news starts spreading with the help of a digital signature, we can find the initiator.	Psychological, Behavioural
	Enabling an Incentive-Based Volunteer Participation Platform	Blockchain-based incentive platform motivates people to volunteer in COVID crisis management by distributing masks, medicines, and daily necessities. Through this mechanism, we can also find who is hoarding products unnecessarily, who is breaking social distancing rules and who is charging extra amounts for needy products. This brings a change in behaviour and relations of people in society.	Interpersonal, Behavioural (as people will stop malpractices)
	Enabling a Secure Donation Platform for Supporters	In order to provide economic help to underprivileged citizens a blockchain-based crowd-funding transparent and highly secured platform is developed. This solves the economic crisis and also reduces cyber crimes.	Economic
	Multi-Robot Collaboration	The multi-robot architecture proposed for combating COVID-19 and other future pandemics work more efficiently with the use of blockchain. The collaboration can be applied towards monitoring the quarantine area, the quarantine E2E delivery system, and the hospital E2E delivery system [50].	Healthcare
	Trusted Sharing of Health Records	A blockchain-based framework constructed using Ethereum tools proposed for addressing the privacy issue and setting up access control through smart contracts, to also help filter accurate information. Blockchain stores only a pointer of the encrypted version of Electronic Health Record [51].	Healthcare
	Security for Drone-Aided Services	Blockchain-enabled secure frameworks were widely sought out during the lockdown for delivery of supply goods as well as healthcare services during the lockdown [44,52].	Economic, Healthcare
AI	Disease Surveillance	Timely surveillance and forecast of novel diseases that can be disastrous for humanity is very crucial. BlueDot’s AI model involving various machine learning and natural language processing tools for forecasting novel diseases. This model tracked the spread of the COVID and forecast its outbreak well before epidemiologists. Many scientists proposed the mode to identify the potentially fatal zoonotic viruses that can cause damage to human beings. Such mechanisms can be used to prepare beforehand with vaccines, drugs, and preventive measures.	Healthcare
	Risk Prediction	There are AI model that can calculate every individual’s vulnerability index based upon factors such as age, travel history, hygiene habits, current health status, pre-existing health conditions, and family medical history that show how much susceptible a person is to COVID-19. The AI-powered predictive model keeps on monitoring COVID infected patients and identifies whether an individual’s condition is serious or who are supposed to shift to ICU wards. There are drug usage databases that are curated using AI by keeping track of particular drug usage by any individual.	Healthcare, Psychological (As risk is predicted it will reduce the stress, strain, fear)
	Medical Diagnosis and Screening	AI-powered cameras with multi-sensors have capabilities to screen people with high body temperature and also recognises their face and their movements. AI-enabled X-rays and CT-scans have saved a lot of time in the diagnosis of COVID. Voice detection backed by AI-tools is used for the screening process in the case of COVID infected patients.	Healthcare
	Virus Modelling and Analysis	AI aid doctors and scientists to understand the structure of the virus and how the virus creates its DNA copies using host cells. After analysis of virus structure, it also helps scientists in tailoring vaccines by suggesting ingredients with the help of huge databases.	Healthcare
	Event Detection System	It involves five modules, which each incorporate different sub-parts of AI for user behaviour changes in online social networks. This involved topic identification and effective analysis, alongside the understanding of the major characteristics of the event [53].	Behavioural
	Busting Fake News	The COVID outbreak and after-effects became the breeding platform for fake news. This fake news consists of a wide range of diverse news that affected human behaviour, mental state, and overall ecosystem of humankind. Big companies such as Google, YouTube, Facebook started using AI to detect fake information and they stopped the circulation of myths, conspiracies, and misinformation on huge social media platforms up to a certain level. These platforms screen and scrap off the content even if there is a slight chance of misinformation.	Behavioural, Psychological
	Enforcing the Lockdown Measures	AI and Computer vision powered infrared cameras are used to scan public spaces and it detects human body temperature, recognises faces and keeps checking human behaviour. In case of violation of social distancing rules, this system sends alerts to relevant authorities.	Behavioural
AI	Host Identification	As there is a novel virus the SARS-CoV-2, which a member of the BetaCov genera of the coronavirus family behind COVID-19, it is very important to understand how this virus is spreading and which are the hosts responsible for spreading this virus. So scientists proposed models, which are based on AI and ML, to detect hosts by using databases of known similar viral genomes.	Healthcare
	Understanding psychological impact	There are a lot of psychological stressors including unemployment, fear of getting infected, hopelessness, helplessness, social isolation, and inadequate psychological support; AI-based models monitor them and predict whether an individual is vulnerable to chronic mental disorders or not [54].	Psychological
	Chatbots	Health chatbots are used for interacting by users for solving some common COVID problems and chatbot monitors and keeps a record of the use by generating reports it smooth outs the process for doctors. Chatbots can also provide details about the user’s location, symptoms, and infection severity score to the users’ doctors or nearby health centres in regular time intervals. Commercial chatbots are used for marketing and after-sales customer support. Some chatbots are used for conversation purposes as through research it is found that people are more comfortable sharing sensitive details with chatbots rather than humans; this could help a person to release stress, anxiety, and provides help to fight against suicidal thoughts and loneliness up to a certain level [55,56,57].	Psychological, Economic, Healthcare
	Telehealth checkup and therapy	There are AI-based algorithms that use NLP (Natural Language Processing) on data collected and by understanding the emotion of individual it provides free preliminary healthcare education, information, and advice related to COVID-19. This model also provides preventive measures, home remedies, interactive counselling, and therapeutic sessions [54].	Psychological, Healthcare
AR/VR	Distance Learning	Real-time online teaching involves the use of audiovisual reality alongside the use of SDN and NVP 5G technologies to provide proper connection [58,59].	Academic
	AR/VR and higher education	higher education, experiential, and collaborative learning is very important. Some concepts cannot be learned without visualising them, so it is crucial to use AR/VR technologies. VR brings such an environment that is not easily accessible to students who is pursuing space studies, archaeological studies, chemical engineering, medical science, aviation related studies, etc. [59,60,61].	Academic
	Factory Automation	In this, AR-based solutions such as remote maintenance solutions contribute to more effective automation due to the implicit reduction in on-site workers. This may help in getting the factories working to their best capacity, in turn helping with the economy [58,62].	Economic
	Virtual Tours	Using the AR/VR solutions of high-quality streaming and holograms alongside the required 5G technologies, e-tourism solutions can be developed, which could also play their part in contributing towards the economic situation. Moreover, it could help mitigate the sense of isolation felt due to the restricted movement, as a result of the pandemic [58,63].	Economic, Psychological
	Customised Surgical Navigation	This is achieved by combining AR, neural networks and customised haptic-enabled surgical tools. The combination provides a novel technique for image-guided COVID-19 lung biopsy with accurate results. On the AR side, a haptic-AR guiding strategy is introduced that accounts for precision and reliable surgery [64].	Healthcare
	Providing Hardware for Diagnostic IoMT	Diagnostic IoMT solution for the COVID-19 telemedicine diagnostic combines customised 5G technology, deep learning algorithms and extended reality (a combination of AR, VR, and MR). The role of extended reality is towards provisioning of the remote surgical plan and rehearse hardware to create a surgical decision system [65].	Healthcare
	Work from Home	To obtain the benefits of a physical workspace in a work from home scenario, VR-based solutions were proposed to impact individual user productivity from home [66] significantly.	Behavioural, Economic
	AR/VR in the Business sector	VR is used to train employees who work in construction, architecture, real estate, and many more, which demands human interaction with real material things. It is used in medical industries to understand and cure patients with visual or cognitive impairments. AR headsets are used by welders, plumbers, mechanics, oil workers, and others who need to head up and hands-free in factory and field works. Customers receive a better shopping experience through AR/VR and they can redesign their home remotely while interacting with interior designers or architects virtually. VR is a boon for manufacturing industries such as aerospace giants as it eases the process of designing and prototyping [67,68,69,70].	Economic
	Tele-immersion	Tele-immersion is an emerging technology that allows users to co-exist virtually in the same space, supporting realistic communication. This proposal could help friends and families reconnect virtually in situations such as social distancing to ease social anxiety and loneliness [71,72].	Social, Psychological

## Data Availability

Not applicable.
